# Epileptic activity on foramen ovale electrodes is associated with sleep and tau pathology in Alzheimer’s disease

**DOI:** 10.1093/brain/awae231

**Published:** 2024-07-11

**Authors:** Astrid Devulder, Greet Vanderlinden, Leen Van Langenhoven, Dries Testelmans, Maarten Van Den Bossche, François-Laurent De Winter, Mathieu Vandenbulcke, Rik Vandenberghe, Tom Theys, Koen Van Laere, Wim Van Paesschen

**Affiliations:** Laboratory for Epilepsy Research, KU Leuven Biomedical Sciences Group, Leuven 3000, Belgium; Department of Neurology, University Hospitals Leuven, Leuven 3000, Belgium; Nuclear Medicine and Molecular Imaging, Department of Imaging and Pathology, KU Leuven Biomedical Sciences Group, Leuven 3000, Belgium; Leuven Biostatistics and Statistical Bioinformatics Centre (L-BioStat), KU Leuven Biomedical Sciences Group, Leuven 3000, Belgium; Laboratory of Respiratory Diseases and Thoracic Surgery (BREATHE), KU Leuven Biomedical Sciences Group, Leuven 3000, Belgium; Department of Pulmonary Diseases, University Hospitals Leuven, Leuven 3000, Belgium; Neuropsychiatry, Department of Neurosciences, Leuven Brain Institute, KU Leuven Biomedical Sciences Group, Leuven 3000, Belgium; Department of Geriatric Psychiatry, KUL University Psychiatric Center (UPC) KU Leuven, Leuven 3000, Belgium; Neuropsychiatry, Department of Neurosciences, Leuven Brain Institute, KU Leuven Biomedical Sciences Group, Leuven 3000, Belgium; Department of Geriatric Psychiatry, KUL University Psychiatric Center (UPC) KU Leuven, Leuven 3000, Belgium; Neuropsychiatry, Department of Neurosciences, Leuven Brain Institute, KU Leuven Biomedical Sciences Group, Leuven 3000, Belgium; Department of Geriatric Psychiatry, KUL University Psychiatric Center (UPC) KU Leuven, Leuven 3000, Belgium; Department of Neurology, University Hospitals Leuven, Leuven 3000, Belgium; Laboratory for Cognitive Neurology, KU Leuven Biomedical Sciences Group, Leuven 3000, Belgium; Research Group Experimental Neurosurgery and Neuroanatomy, KU Leuven Biomedical Sciences Group, Leuven 3000, Belgium; Department of Neurosurgery, University Hospitals Leuven, Leuven 3000, Belgium; Nuclear Medicine and Molecular Imaging, Department of Imaging and Pathology, KU Leuven Biomedical Sciences Group, Leuven 3000, Belgium; Division of Nuclear Medicine, University Hospitals Leuven, Leuven 3000, Belgium; Laboratory for Epilepsy Research, KU Leuven Biomedical Sciences Group, Leuven 3000, Belgium; Department of Neurology, University Hospitals Leuven, Leuven 3000, Belgium

**Keywords:** epileptiform activity, mesial temporal lobe, neuronal hyperexcitability in Alzheimer’s disease, polysomnography, scalp-intracranial EEG, ^18^F-MK6240 tau-PET

## Abstract

Both sleep alterations and epileptiform activity are associated with the accumulation of amyloid-β and tau pathology and are currently investigated for potential therapeutic interventions in Alzheimer's disease. However, a bidirectional intertwining relationship between sleep and neuronal hyperexcitability might modulate the effects of Alzheimer's disease pathology on the corresponding associations. To investigate this, we performed multiple day simultaneous foramen ovale (FO) plus scalp EEG and polysomnography recordings and acquired ^18^F-MK6240 tau PET-MR in three patients in the prodromal stage of Alzheimer's disease and in two patients with mild and moderate dementia due to Alzheimer's disease, respectively. As an eligibility criterion for the present study, subjects either had a history of a recent seizure (*n* = 2) or subclinical epileptiform activity (SEA) on a previous scalp EEG taken in a research context (*n* = 3). The ^18^F-MK6240 standard uptake value ratio (SUVR) and asymmetry index (AI) were calculated in *a priori*-defined volumes of interest. Linear mixed-effects models were used to study associations between interictal epileptiform discharges (IEDs), polysomnography parameters and ^18^F-MK6240 SUVR.

Epileptiform activity was bilateral but asymmetrically present on FO electrodes in all patients and ≥95% of IEDs were not visible on scalp EEG. In one patient, two focal seizures were detected on FO electrodes, both without visual scalp EEG correlate. We observed lateralized periodic discharges, brief potentially ictal rhythmic discharges and lateralized rhythmic delta activity on FO electrodes in four patients. Unlike scalp EEG, intracranial electrodes showed a lateralization of epileptiform activity. Although the amount of IEDs on intracranial electrodes was not associated to the ^18^F-MK6240 SUVR binding in different volumes of interest, there was a congruent asymmetry of the ^18^F-MK6240 binding towards the most epileptic hemisphere for the mesial (*P =* 0.007) and lateral temporal cortex (*P =* 0.006). IEDs on intracranial electrodes were most abundant during slow wave sleep (SWS) (92/h) and non-REM sleep 2 (N2, 81/h), followed by non-REM sleep 1 (N1, 33/h) and least frequent during wakefulness (17/h) and REM sleep (9/h). The extent of IEDs during sleep was not reflected in the relative time in each sleep stage spent [REM% (*P* = 0.415), N1% (*P* = 0.668), N2% (*P* = 0.442), SWS% (*P* = 0.988)], and not associated with the arousal index (*P* = 0.317), apnoea-hypopnoea index (*P* = 0.846) or oxygen desaturation index (*P* = 0.746). Together, our observations suggest a multi-directional interaction between sleep, epileptiform activity and tau pathology in Alzheimer's disease.

## Teaser

The mesial temporal lobe (MTL) is affected early in Alzheimer's disease and is prone to hyperexcitability. Devulder *et al.* show that MTL epileptic activity detected with intracranial electrodes is modulated by sleep, and that lateralized epileptic activity is congruent with asymmetric temporal lobe tau pathology.

## Introduction

In Alzheimer's disease (AD), extracellular amyloid-β plaques start to accumulate in the neocortex 15–20 years before the onset of cognitive decline. This is followed by the deposition of intraneuronal neurofibrillary tangles composed of hyperphosphorylated tau, spreading from the mesial temporal lobe to the neocortex, which is associated with the onset of neurodegeneration and clinical symptoms.^1,2^

Amyloid-β and tau pathology are linked to network hyperexcitability.^3-10^ A current hypothesis is that amyloid-β disrupts the excitatory-inhibitory balance and triggers epileptogenesis, which stimulates the release and spread of tau from the transentorhinal cortex through mesial temporal lobe to the neocortical brain regions, which is associated with the onset of cognitive symptoms. This results in neuronal loss and altered neuronal signalling, which in turn promotes epileptiform activity, creating a vicious circle.^3,4,10,11^ Mesial temporal epileptiform activity has been associated with tau pathology^10,12^ and cognitive symptoms^13^ and was hence, the focus of our work. Epileptiform activity in AD is associated with a more accelerated cognitive decline.^14,15^ Counteracting neuronal hyperexcitability with anti-seizure medication (ASM) can have beneficial effects on cognitive function, leading to potential disease-modifying opportunities.^16,17^

In addition, there is emerging evidence for a bidirectional link between AD pathology and sleep.^18-20^ Sleep alterations and obstructive sleep apnoea (OSA) have been associated with amyloid-β and tau pathology in cognitively impaired^21-24^ and intact subjects.^25-34^ Sleep alterations are inherent to increasing age, however, patients with AD suffer from more pronounced or distinct sleep disruptions. These include more fragmented sleep, lower sleep efficacy, decreases in slow wave sleep (SWS) and REM sleep as well as co-morbid OSA.^24,35-39^ Sleep alterations tend to aggravate during the disease course and have been associated with cognitive functioning.^24,35,39^ Therefore, interventions for optimizing sleep are currently investigated as potential therapeutic opportunities.^40^

Sleep and epilepsy have a complex intertwining relationship.^41^ High amplitude slow waves during non-REM (N) sleep are the main driver of interictal epileptic activity.^42^ Epileptiform activity, in turn, has arousing effects during sleep.^43^ A study in *Drosophila* flies demonstrated that chronic sleep deprivation exacerbated amyloid-β induced neuronal hyperexcitability.^44^ This suggests an underlying link between sleep alterations and neuronal hyperexcitability, as well as reciprocal modulations, during AD progression. Yet, it still needs to be determined how sleep and hyperexcitability interact in human AD and if a more integrated approach could optimize therapeutic interventions. In previous scalp EEG studies, epileptiform activity was mainly detected in the temporal lobes during SWS and non-REM 2 (N2) sleep.^14,15,45-47^ However, scalp EEG has limited sensitivity in detecting temporal lobe epileptiform activity,^48,49^ which is reflected in the variable prevalence and overall low frequency of epileptiform discharges reported in previous studies.^14,15,45-47^ Foramen ovale (FO) electrodes may be a more sensitive, yet more invasive, surgical option than scalp EEG to measure mesial temporal epileptiform activity.^50,51^

Here, we performed combined simultaneous FO-scalp EEG with polysomnography (PSG) and acquired ^18^F-MK6240 tau PET-MR in five patients with AD from our initial study cohort^45^ with subclinical epileptiform activity (SEA) on scalp EEG or a seizure history to investigate the interaction between sleep, neuronal hyperexcitability and tau pathology.

## Materials and methods

### Participants

Five patients with AD [three males, median age 76 years (65–79 years)], who met the National Institute of Aging-Alzheimer's Association criteria for AD^52-54^ (Supplementary material, ‘Methods’ section 1.1 and Supplementary Table 1) were included. They were selected from our initial observational cross-sectional study cohort,^45^ which consisted of 41 patients with AD, without formal epilepsy diagnosis, who underwent an overnight ambulatory scalp EEG between September 2019 and December 2022. In this cohort, SEA was detected in 14 patients (34%), of which two had a history suspect for seizures which was assessed with the Reutens Questionnaire for Clinical Seizure diagnosis.^55^ Furthermore, there was one patient with a positive questionnaire, although no epileptiform activity was detected on scalp EEG. To subsequently enrol in the present study, inclusion criteria were the presence of SEA on scalp EEG or a probable seizure history. In total, five patients participated in this study between May 2022 and February 2023. Three were recruited from the memory clinic and two from the geriatric psychiatry department at the University Hospitals Leuven. Informed consent was obtained from all patients or their assigned caregiver. The study was approved by the Ethical Committee of the University Hospitals Leuven (clinical.trials.gov NCT03617497).

### Foramen ovale electrode placement

The placement of bilateral four-contact FO electrodes (AdTech) was performed in the operating room under general anaesthesia (dorsal decubitus, endotracheal tube). A postoperative CT scan was performed to confirm correct positioning and exclude intracranial haemorrhage (Supplementary Fig. 1).

### EEG and polysomnography recording

All participants underwent continuous video FO-scalp EEG monitoring for three to six consecutive days and nights. Scalp electrodes were placed according to the International 10–20 system with additional lower temporal line electrodes (F10, T10, P10, F9, T9, P9),^56^ ECG, electrooculogram (EOG) 1/2 and chin EMG. Seizures, interictal epileptiform discharges (IEDs), lateralized rhythmic delta activity (LRDA), lateralized periodic discharges (LPDs) and brief potentially ictal rhythmic discharges (BIRDs) were evaluated both on scalp EEG and FO electrodes conform the International Federation for Clinical Neurophysiology (IFCN) criteria and American Clinical Neurophysiology Society (ACNS) criteria by two readers (A.D. and W.V.P.), with 3 and 40 years of experience, respectively.^57,58^ In case of disagreement, consensus was reached after open discussion. The Spike Detector of the Persyst 14 EEG software (Persyst) was used to detect IEDs on both intracranial electrodes and scalp EEG.^59,60^ The annotations of the Persyst software were visually reviewed, discarded if they did not met the IFCN criteria,^58^ and quantified per hour. Small sharp spikes of sleep and wicket spikes were not included as epileptiform activity. A detailed description of the EEG review process is available in the Supplementary material, ‘Methods’ section 1.2. We determined the most epileptogenic hemisphere based on FO electrodes by a visual assessment of lateralization of overall epileptiform activity and by calculating the asymmetry index (AI) of IEDs, respectively. The AI of IEDs was calculated towards the right hemisphere with the following formula: AI = (IEDs/h right – IEDs/h left) × 200 / (IEDs/h right + IEDs/h left). For each overnight PSG additional sensors included nasal flow (NAF2P), thoracic and abdominal resistance bands, finger pulse oximetry and EMG of one leg. Sleep was scored according to the American Academy of Sleep Medicine (AASM) scoring rules version 2.6^61^ by two readers. Electrodes P9 and P10 were used as reference electrodes instead of A1 and A2. Apnoeas were scored on the flow signal on NAF2P. The apnoea-hypopnoea index (AHI) was defined as the total number of apnoeas and hypopnoeas per hour of sleep and the oxygen desaturation index (ODI) was defined as the number of desaturations ≥3% per hour of sleep. The arousal index was calculated as the total number of arousals and awakenings per hour on total sleep time (TST), without distinction between respiratory-related and other arousals. In order to study the associations of interest between sleep and epileptic activity, the EEG and PSG parameters were quantified from lights off until lights on (9 h ± 1 h). Recordings were obtained with BrainRT EEG (OSG, Kontich) with a sampling rate of 256 Hz.

### PET and MR data acquisition and reconstruction

All five participants underwent ^18^F-MK6240 PET-MR on a 3 T Signa scanner (GE Healthcare), with a static acquisition 90–120 min post injection (injected activity 160.7 ± 15.4 MBq). 3D volumetric T1-weighted MR images were acquired simultaneously with PET acquisitions using a vendor-supplied 32-channel high resolution brain phased array head coil. Reconstructed ^18^F-MK6240 5-min frames were corrected for motion and averaged. A partial volume correction was performed using a region based voxelwise algorithm.^62^ Standard uptake value ratios (SUVR) for different brain volumes-of-interest (VOIs) were calculated, using the inferior cerebellar grey matter as reference region. The CAT12 toolbox of SPM12 (Statistical Parametric Mapping, Wellcome Trust Centre for Neuroimaging, University College, London, UK) was used for segmentation of T1-weighted MR images. According to the normalization of CAT12, six *a priori*-defined, composite, bilateral VOIs were quantified based on the Neuromorphometrics Atlas in native space for each participant: mesial temporal cortex (entorhinal cortex, parahippocampal gyrus, hippocampus and amygdala), lateral temporal cortex, frontal cortex, parietal cortex, occipital cortex and cingulate cortex. An AI for ^18^F-MK6240 SUVR was calculated relative to the most epileptogenic hemisphere as AI SUVR = (SUVR ipsilateral – SUVR contralateral) × 200 / (SUVR ipsilateral + SUVR contralateral).

### Statistical analysis

The proportion of IEDs on scalp EEG and FO electrodes was assessed for each night for each participant. Depending on normality (Shapiro-Wilk test, α ≤ 0.05), a one sample *t*-test or Wilcoxon signed-rank test was used to verify if the AI of the IEDs and the AI of the SUVR was different from zero. For each night, IEDs/FO electrodes were calculated per vigilance state, divided by the numbers of hours spent in that particular state. To study the associations of interest in relation to IEDs on FO electrodes and PSG parameters for each night, we used linear mixed effects models with the IEDs/h on FO electrodes as outcome variable, one fixed effect variable and if possible a random effect for intercept and slope per patient was added, if not, only a random intercept was modelled. These models allowed to account for intra-individual variability between consecutive nights for each patient. Before applying the model, the outcome was log-transformed. The resulting fixed effect slopes are presented in Supplementary Table 2. These resulting associations should be interpreted as marginal, average effects, and should not be used for patient-specific predictions. To estimate the differences in IEDs/h between different vigilances states, the outcome was transformed with a square root. A mixed model with vigilance state as fixed effect and intercept and vigilance state as random effects, was then used to estimate the outcome for each vigilance state. The differences between vigilance states were then estimated using a non-linear estimation method. The same linear mixed regression approach was used to study the associations of interest in relation to IEDs on FO electrodes and ^18^F-MK6240 SUVR. The outcome was log transformed, except for the AI of IEDs on FO electrodes. Since this was an explorative study involving testing of comparisons which are regarded as hypotheses for further investigation, we did not perform correction for multiplicity testing in order not to increase the type II error. All tests were two-sided and assessed at a significance level of 5% and all confidence intervals were set at 95%. Analyses were performed using SAS software 9.4 of the SAS System for Windows (Copyright ©2002 SAS Institute Inc., Cary, NC, USA) and R studio (R Studio: Integrated Development Environment for R. Posit Software, PBC, Boston, MA. http://www.posit.co/).

## Results

### Clinical characteristics

One patient in a moderate dementia stage due to AD (Patient 1), one patient in a mild dementia stage due to AD (Patient 2) and three patients in a prodromal stage of AD (Patients 3–5), were included. All participants were white Caucasian. One participant (Patient 5) was diagnosed with early onset AD. Two patients had suspected seizures: the first patient (Patient 1) had confusional nocturnal awakenings and experienced episodes where she became pale, hyperventilated, followed by a brief loss of consciousness. During previous prolonged ECG monitoring, she had four episodes of bradycardia up to 43 beats/min without clinical symptoms. On prior scalp EEG, performed per study protocol,^45^ we did not detect epileptiform activity, albeit no episodes were reported by the patient or caregiver during the recordings. This patient was recruited from the memory clinic UZ Leuven. She was in a moderate dementia stage, took mirtazapine 15 mg daily and required haloperidol (2–5 mg) during hospitalization. The second patient (Patient 2), recruited from the geriatric psychiatry department, was in a mild dementia stage. He experienced five episodes of staring, automatisms and dystonia of the right arm during the past months. Prior scalp EEG, conform study protocol,^45^ revealed sporadic IEDs on frontal electrodes (six IEDs on F9, one IED on F10). After the patient signed the informed consent for further registration with FO electrodes, he had a nocturnal tonic-clonic seizure. Levetiracetam 500 mg twice daily was initiated, which was decreased and stopped during the intracranial recordings. Clinical characteristics are summarized in Table 1.

**Table 1:** Clinical characteristics

Pt	Sex	Age	Duration of symptoms (years)	MMSE	CDR	BMI	Caffeine^a^	Ethyl^a^	Previous overnight ambulatory EEG findings	Reutens questionnaire	Comorbidities	Medication
1	F	73	5	12/30	2	19	2.5	1	Normal	Positive: episodes of pallor, hyperventilation and sudden loss of consciousness; nocturnal confusional awakenings	Hypertension	Bisoprolol-hydrochlorothiazide Mirtazapine Haloperidol during hospitalization
2	M	77	3	26/30	0.5	25	3	1	7 spikes (6×F9, 1×F10)	Positive: episodes of staring, automatisms and dystonia of the right arm	Presbyacusis	Levetiracetam (decreased in dose during hospitalization) Aspirin in primary prevention Donepezil
3	F	76	3	29/30	0.5	25	2	1	1 spike (1×T9)	Negative	Hypertension Urge incontinence	Pantoprazole Lisinopril Nebivolol-hydrochlorothiazide Mirabegron Donepezil
4	M	79	2	29/30	0.5	24	2	0	3 spikes (3×F8)	Negative	Hypertension Prostate carcinoma R/Radiotherapy + hormone therapy (curative) Mild depressive symptoms	Nebivolol Donepezil Escitalopram Amlodipine Tamsulosin
5	M	65	2	29/30	0.5	25	3	2	Temporal LRDA left	Negative	Hypertension Hypercholesterolemia Uvulopalatopharyngoplasty Gout Chronic kidney disease of unknown origin	Quinapril Donepezil Verapamil Simvastatin Losartan-hydrochlorothiazide

BMI = body mass index; CDR = Clinical Dementia Rating Scale; F = female; LRDA = Lateralized rhythmic delta activity; M = male; MMSE = Mini-Mental State Examination; Pt = patient.

^a^Numbers represent units per day.

### The discrepancy between epileptiform activity on scalp EEG and intracranial electrodes

**Figure 1:**
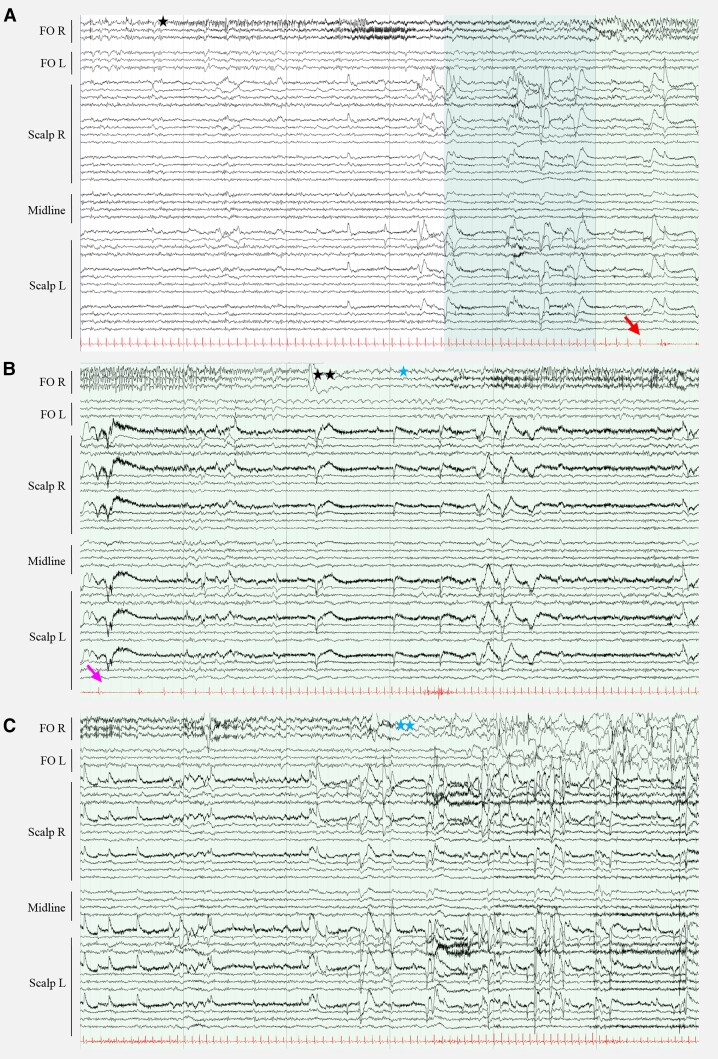
**Mesial temporal lobe seizures.** Two unilateral nocturnal seizures were visible on the right foramen ovale electrode (FO R) in Patient 1. (**A**) The first seizure started during non-REM sleep 1 (N1) with paroxysmal fast activity on FO R (black star). Clinical arousal (dark shaded area) with transition to wakefulness (lighter shaded area). Ictal bradycardia (red arrow) was present. (**B** and **C**) Continuous ictal activity on FO R, evolving to more rhythmic spike-wave activity with bradycardia progressing to a cardiac pause of 5 s (pink arrow) with an abrupt end after 79 s (two black stars). The second seizure started during wakefulness, 8 s after the first seizure ended (blue star), with paroxysmal fast activity evolving to a rhythmic spike-wave pattern and ending after 58 s (two blue stars). The second seizure had no clinical correlate. Scalp EEG did not reveal epileptic activity. Each panel represents continuous EEG epochs of 60 s. Bipolar montage. Filters at 0.53–30.0 Hz. Sensitivity 30 µV for FO electrodes, 70 µV for scalp EEG.

**Figure 2:**
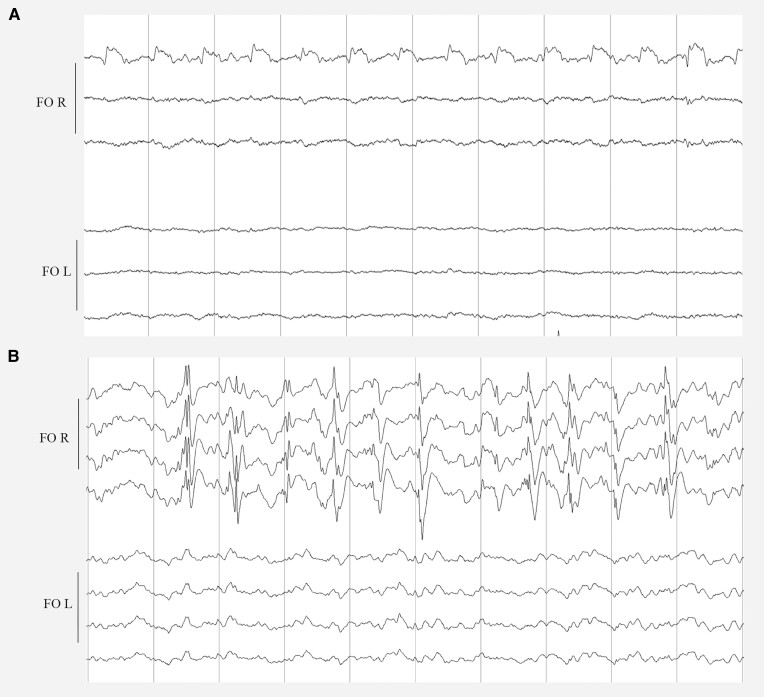
**Lateralized periodic discharges (LPDs) on foramen ovale electrodes**. (**A**) In Patient 1, LPDs on the right foramen ovale electrode (FO R) consisted of spikes, which occurred at ±1 Hz. (**B**) In Patient 2, LPDs on FO R consisted of spikes and polyspikes, which occurred at a fluctuating frequency of 1–2 Hz, with no evidence of evolution. Each panel represents a 10-s epoch. Filters at 0.53–30.0 Hz. In Patient 1, a bipolar montage with a sensitivity of 30 µV is shown; in Patient 2, a reference montage with a sensitivity of 100 µV is shown.

**Figure 3:**
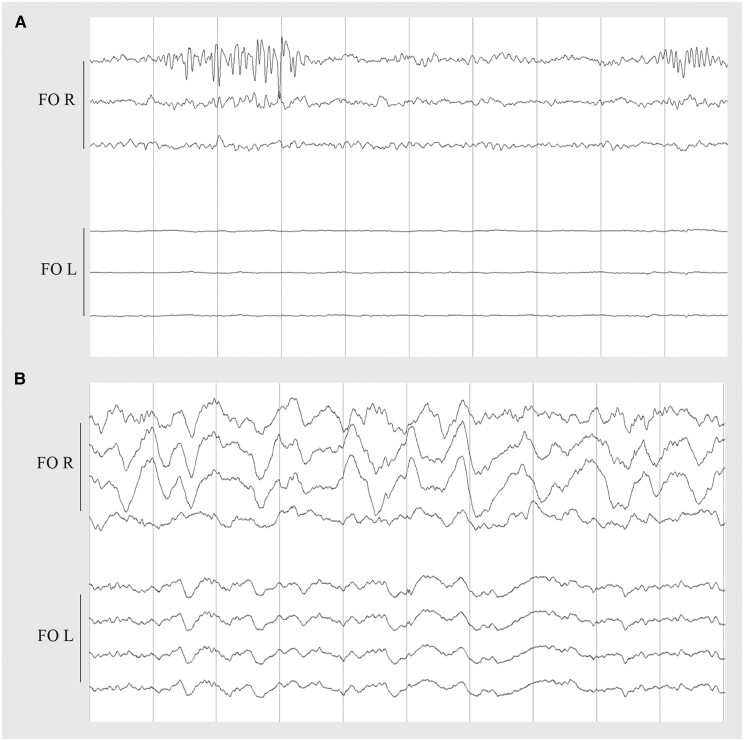
**Brief potentially ictal rhythmic discharges (BIRDs) and lateralized rhythmic delta activity (LRDA) on foramen ovale electrodes.** (**A**) BIRDs on right foramen ovale electrode (FO R) in Patient 5: two episodes of ±11 Hz, sharply contoured, high amplitude waves, lasting 2.5 and 1 s, respectively. (**B**) LRDA on FO R in Patient 5. Each panel represents a 10-s epoch. Bipolar montage in **A**, reference montage in **B**. Sensitivity at 150 µV, filters at 0.53–30.0 Hz.

**Figure 4:**
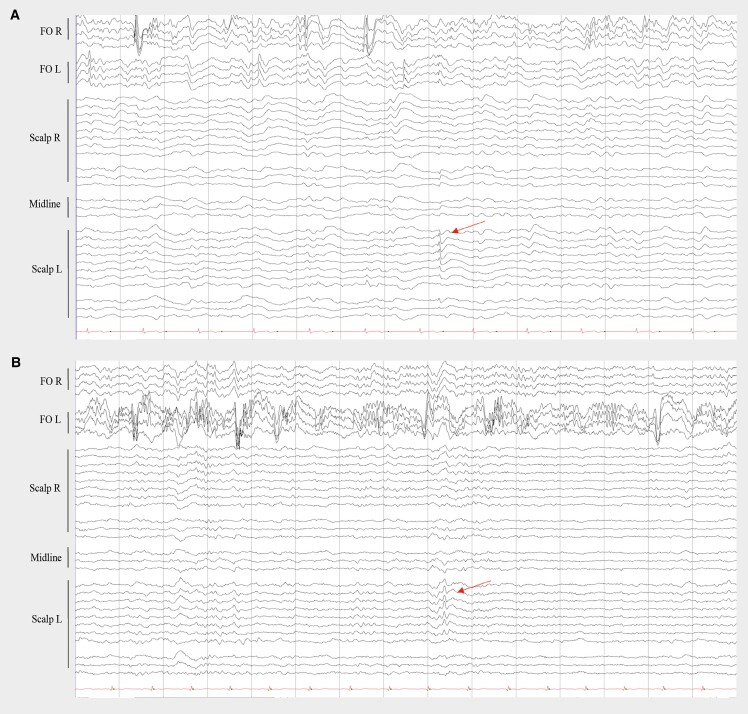
**Interictal epileptiform discharges (IEDs) on scalp EEG and foramen ovale electrodes.** (**A**) In Patient 2, IEDs can be seen on the right foramen ovale electrode (FO R) without scalp EEG correlate. One IED on scalp left (L, arrow) without corresponding IED on FO electrodes. (**B**) In Patient 3, IEDs are abundant on the left FO electrode (FO L) with only one visible correlate on scalp EEG (arrow). Each panel represents a 15-s epoch. Reference montage. Sensitivity at 100 µV for FO electrodes, 70 µV for scalp EEG. Filters at 0.53–30.0 Hz.

We measured two unilateral nocturnal seizures on the right FO electrode without scalp EEG correlate in one patient. The first seizure was associated with an awakening from N1 and ictal bradycardia with sinus arrest lasting 5 s. The second seizure started during wakefulness and had no clinical correlate (Fig. 1A–C). We registered IEDs on FO electrodes in all patients, which were frequently (10%–49% of EEG recording) present in two patients, occasionally (1%–9%) present in two patients and rarely (<1%) visible in one patient. In three patients, the IEDs on FO electrodes appeared as periods of non-evolving LPDs between 0.5–2 Hz of very-brief (<10 s) to intermediate duration (<9.9 min) (Fig. 2), while we did not identify LPDs on scalp EEG. We observed lateralized bursts of sharply contoured rhythmic activity in the alpha or beta range on FO electrodes, which fulfilled the criteria of BIRDs^57^ in three patients (Fig. 3A), and in two patients occasional LRDA was present on FO electrodes (Fig. 3B). In four patients, we detected few IEDs on scalp EEG, whereas one subject had only two spikes over a period of four nights. The median frequency of spikes detected on scalp EEG over the whole night in these four patients was seven (range 0–41 spikes). In one patient we identified rare very brief periods of temporal LRDA on scalp EEG (Supplementary Fig. 2). We quantified IEDs/h for both scalp EEG and FO electrodes and the median ratio of IEDs/h on scalp versus FO electrodes was between 0% and 5% for all participants, with often no scalp correlate for the IEDs on FO electrodes. However, we also identified IEDs on scalp EEG without corresponding IEDs on FO electrodes (Fig. 4). Epileptiform activity, although present on both FO electrodes, was lateralized to one hemisphere in all patients. More specifically, there was a right-sided predominance of epileptiform activity in four patients and a left-sided predominance in one patient. In contrast, epileptiform activity on scalp EEG, if detected at all, was not remarkably lateralized. Moreover, in one patient the lateralization of IEDs and LRDA on scalp EEG was opposite to the predominant side of the epileptiform activity on intracranial electrodes. Scalp and intracranial EEG data are summarized in Table 2.

**Table 2:** Scalp and Foramen ovale EEG data

		Scalp EEG			Foramen ovale electrodes			Median ratio of IEDs scalp versus FO in % [range]
Pt	Nights recorded	Background	Epileptiform activity	Mean AI [range] of IEDs on scalp EEG towards R hemisphere	Background	Epileptiform activity	Mean AI [range] of IEDs/FO towards R hemisphere	
1	3	Alpha range.Symmetric.	No IEDs	–	Symmetric	2 seizures right; Rare blunt LPDs right (1 Hz) brief to intermediate duration; IEDs median 22/h [range 18/h–24/h], mean 21/h ± 3/h	17 [−12 to 35]	0%
2	6	Alpha range. Mild slowing right hemisphere.	IEDs on F7, F8, F9, F10, Fp2, T9, T10; median 5/h [range 1/h–14/h], mean 6/h ± 5/h	−84 [−175 to 100]	Marked asymmetry with right-sided slowing and higher voltage	Rare LPDs right-sided (1.5–2 Hz) brief to intermediate duration; Bursts of sharply contoured rhythmic activity in the alpha range, resembling BIRDs; IEDs median 94/h [range 24/h–249/h], mean 110/h ± 81/h	93 [44 to 149]*	5% [1–33%]
3	3	Alpha range. Symmetric.	IEDs on F7, F10, P10, T5, T9, T10; median 1/h [range 0.9/h–1.3/h], mean 1/h ± 0.2/h	−45 [−80 to 22]	Marked asymmetry with left-sided slowing and higher voltage	Occasional (semi-) rhythmic delta activity (LRDA) with superimposed fast activity left-sided brief to intermediate duration; Bursts of high-frequency rhythmic activity in the beta range, resembling BIRDs; IEDs median 31/h [range 30/h–107/h], mean 56/h ± 45/h	−178 [−189 to −158]*	3% [1–4%]
4	4	Generalized beta. Symmetric.	Two IEDs on F8 and T9; median 0/h	–	Mild asymmetry with left-sided limited slower activity	IEDs median 5/h [range 1/h–7/h], mean 5/h ± 2/h	66 [45 to 98]*	1% [0–2%]
5	4	Alpha range. Mild slowing left hemisphere.	IEDs on F7, F9, T9; Median 0.4/h [0.3/h–0.5/h], mean 0.4/h ± 0.1/h Rare very brief periods of temporal LRDA left	−200*	Marked asymmetry with right sided slowing	Occasional LPDs right sided (0.5–2 Hz) very brief to brief duration; Occasional rhythmic delta activity right-sided (LRDA); Bursts of sharply contoured rhythmic activity in the alpha range, resembling BIRDs; IEDs median 88/h [range 38/h–142/h], mean 89/h ± 57/h	118 [79 to 164]*	1% [0–1%]

AI = asymmetry index; BIRDs = brief potentially interictal rhythmic discharges; FO = foramen ovale; IEDs = interictal epileptiform discharges, LPDs = lateralized periodic discharges; LRDA = lateralized rhythmic delta activity; Pt = patient; R = right.

^*^ *P*-values < 0.05 for one sample *t*-test.

### Mesial temporal epileptic activity is highest during slow wave sleep

Following the observation that mesial temporal epileptiform activity is underestimated on scalp EEG^48,49^ we studied possible associations between epileptiform activity on intracranial electrodes and sleep. Since we detected only two seizures, we focused on associations between IEDs and PSG parameters. On FO electrodes, the amount of IEDs/h were highest during SWS (mean = 92/h; CI, 34/h–178/h) and N2 (mean = 81/h; CI, 26/h–165/h), followed by N1 (mean = 33/h; CI, 4/h–91/h). The number of spikes on FO electrodes was lowest during wakefulness (mean = 17/h; CI, 0/h–63/h) and during REM (mean = 9/h; CI, 0/h–46/h). The frequency of IEDs during SWS was significantly higher compared to all other vigilance states except for N2 (SWS versus N1, *P* = 0.011; SWS versus N2, *P* = 0.697; SWS versus REM, *P* = 0.002; SWS versus W, *P* = 0.002). The IEDs/h during N2 were significantly higher compared to REM (*P* = 0.008) and wakefulness (*P* = 0.013) but not compared to N1 (*P* = 0.054), and the IEDs/h during N1 were significantly higher compared to REM (*P* = 0.028) but not compared to wakefulness (*P* = 0.093). The amount of IEDs/h during wakefulness was not significantly higher compared to REM sleep (*P* = 0.190). The median REM% ranged from 4% to 25% between participants with a large intra-individual variability and was not statistically significantly associated with the amount of IEDs/h TST on FO electrodes (*P* = 0.415). One intracranially detected seizure during N1 was associated with arousal, but the arousal index on TST showed no correlation with the amount of IEDs/h TST on intracranial electrodes (*P* = 0.317). We registered moderate to severe OSA over the different nights in four participants. One participant had no or mild OSA and only rare epileptiform activity. Despite this, the amount of IEDs/h TST detected on FO electrodes was not statistically significantly associated with the AHI on TST (*P* = 0.846) or ODI on TST (*P* = 0.764) and in both non-REM and REM separately, the amount of intracranially registered IEDs/h was not correlated to the AHI in the corresponding sleep stages (*P* = 0.666; *P* = 0.777) (Supplementary Table 2). Individual PSG parameters are available in Supplementary Table 3.

### Asymmetric mesial temporal neuronal hyperexcitability lateralizes to increased temporal lobe tau pathology

**Figure 5:**
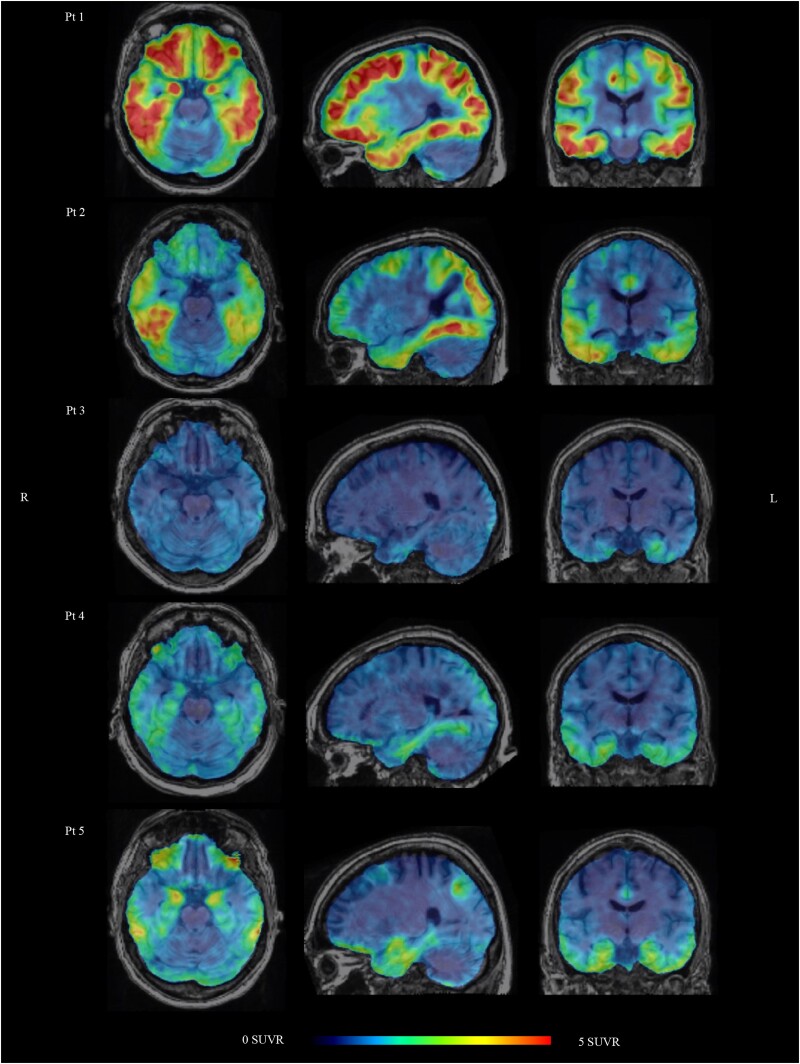
**^18^F-MK6240 orthogonal PET images through the mesial temporal cortex, overlaid on MR.** All patterns are consistent with Alzheimer’s disease. The ^18^F-MK6240 binding in the mesial and lateral temporal lobe shows a lateralization to the epileptogenic hemisphere: right-sided for Patients 1, 2, 4 and 5; left-sided for Patient 3. SUVR = standardized uptake value ratio.

All participants had increased mesial temporal ^18^F-MK6240 tracer uptake (Fig. 5). Since epileptiform activity on FO electrodes was lateralized to one hemisphere in all patients, we searched for asymmetry in tau pathology. The ^18^F-MK6240 AI showed significant lateralization to the most epileptogenic hemisphere for the mesial temporal cortex (mean AI = 17.9; CI, 8.0–27.8, *P =* 0.007) and lateral temporal cortex (mean AI = 18.4; CI, 8.8–28.0, *P =* 0.006); no statistically significant lateralization was found for the cingulate cortex (mean AI = 17.4; CI −1.0–35.8, *P =* 0.063), frontal cortex (mean AI = 6.6; CI, −0.1–13.3, *P =* 0.052) and parietal cortex (mean AI = 24.8; CI, −1.8–51.4, *P =* 0.061). The extent of asymmetry of IEDs on FO electrodes was not associated with the AI of the ^18^F-MK6240 SUVR in different VOIs (Supplementary Fig. 3) and the amount of IEDs on FO electrodes was not associated with the ^18^F-MK6240 tracer binding in the different VOIs (Supplementary Fig. 4).

## Discussion

This cross-sectional exploratory study, using simultaneous FO-scalp EEG/PSG and ^18^F-MK6240 PET-MR, shows the underreported presence of epileptiform activity in AD and how it is associated with sleep and tau pathology *in vivo*. Our findings demonstrate that epileptiform activity in AD entails a whole spectrum consisting of IEDs, rhythmic and periodic patterns, BIRDs and (sub)clinical seizures, which can already be present in the prodromal AD stage irrespective of a seizure history. The frequency of epileptiform activity on FO electrodes varied across all patients, but was consistently under detected on scalp EEG as temporal lobe seizures were not discernible on scalp EEG and ≤5% of IEDs, which were most abundant during SWS, were detected on scalp EEG. Unlike scalp EEG, intracranial electrodes revealed a bilateral but asymmetric representation of epileptic activity, congruent with the dominant side of predominant temporal lobe tau pathology.

A remarkably consistent finding from the present study is the discrepancy between scalp EEG and intracranial recordings. The lack of visual scalp EEG correlate of seizures, rhythmic patterns and IEDs that could be recorded intracranially highlights the underestimated *prevalence* of epileptiform activity in AD, but even more so the ‘frequency’ of epileptiform activity, if detected at all.^15,49^ Although the frequency of intracranially registered IEDs in our sample was lower compared to the IED frequency reported by Lam *et al.,*^49^ the ratio wherein ≤5% of IEDs on FO electrodes was detected on scalp EEG was in line. A different patient selection and method, where they performed a manual quantification of randomly sampled EEG segments, could serve as a potential explanation.

One scalp-negative hippocampal seizure was associated with ictal bradycardia and asystole.^63,64^ The main aetiology of transient loss of consciousness in dementia is syncope, which has an unexplained cause in around 13%.^65^ Although this patient had an ictal asystole of 5 s without syncope, we suggest that transient loss of consciousness in patients with AD could be a manifestation of ictal bradycardia and asystole. Vossel *et al.*^66^ described five patients with AD and epilepsy, who had a pacemaker implantation for an ictal bradycardia syndrome. Hence, transient loss of consciousness in the elderly with AD warrants screening for epileptic activity and ictal bradycardia syndrome.^66^

Interestingly, besides seizures and IEDs, we documented periods of LPDs, LRDA and BIRDs on intracranial electrodes. These patterns, when found on scalp EEG are associated with an increased risk for seizures in critically ill patients and with refractory epilepsy in the non-critically ill.^57,67,68^ Although, the clinical and pathophysiological significance of these intracranial recording patterns still needs to be determined in AD, our findings support their association with hyperexcitability^47,67,68^ and increased seizure risk, as documented in our first patient, who also had LPDs. The presence of very brief periods of temporal LRDA on scalp EEG, which appeared independently of LRDA on FO electrodes, is likely related to an underlying cortical dysfunction, linked to altered network excitability as has previously been suggested.^47^ However the lateralizing value of temporal LRDA in AD might be questioned since the detected side in our fifth patient was opposite to the predominant epileptic hemisphere on FO electrodes.^69,70^

This intracranial EEG-PSG study allowed to examine associations between sleep and epileptiform activity. Interictal epileptiform activity increased during sleep, particularly during SWS, a period critical for memory consolidation.^71^ Through disruption of the physiological coupling between slow oscillations, sleep spindles and hippocampal ripples required for hippocampal-neocortical information transfer,^71,72^ mesial temporal IEDs interfere with both short^73^ and long-term memory consolidation during sleep, with the largest effect if the number of seizures is low.^74^ Mesial temporal lobe seizures and interictal mesial temporal epileptic activity are also related to arousals from sleep by effects on the sleep-wake structures.^43,75,76^ Therefore, neuronal excitability can affect the formation and stabilization of synapses formed after learning during non-REM and REM sleep, respectively.^72,77^ Indeed, the electrographic seizure we recorded during sleep was associated with arousal. Although no direct association between the amount of IEDs and arousal index was found, a sleep disrupting effect of IEDs cannot be ruled out since we did not study the timing between IEDs and arousal or the impact on specific frequencies through the different sleep stages.^78^ The highest prevalence of IEDs during SWS followed by N2 is in line with the observations of Horvath *et al*.^14^; however, this differs from previous scalp EEG studies^45-47^ wherein IEDs were most frequently reported during N2. Albeit the difference between the burden of IEDs during SWS and N2 reported in current study is small, possible explanations for discrepancy with previous work are the rather small sample sizes of different studies, the methods used to score sleep, and the fact we specifically counted IEDs on FO electrodes. The invisibility of mesial temporal IEDs on scalp EEG in AD,^49^ hence complicates a direct comparison to previous scalp EEG studies. In OSA, sleep disruption and recurrent oxygen desaturation were associated with increased epileptiform activity.^79^ Correlations between AHI and epileptiform activity on scalp EEG and a reduction of seizures and IEDs after a period of continuous positive airway pressure (CPAP) treatment, were reported in small studies in patients with epilepsy and comorbid OSA.^80-84^ In our previous cross-sectional study we measured a higher AHI on TST in AD patients with SEA on scalp EEG.^45^ In the present study, the one patient with no or mild OSA during the recorded nights, had rare IEDs on FO electrodes. It was, therefore, rather surprising that we did not find a significant association between the amount of IEDs and AHI or ODI on TST, respectively. Despite the fact our study only consists of five patients, which means we don't have much statistical power to detect associations, one potential explanation is that studies between OSA and epilepsy^80-84^ were limited to scalp EEG and were conducted in younger patients with different types of epilepsy, without AD biomarkers. Here the effect between sleep and neuronal hyperexcitability could be modulated by AD pathology. Although the exact mechanisms still need to be elucidated, it is likely that OSA through chronic sleep disruption, hypoxic burden and impaired protein clearance can indirectly influence neuronal hyperexcitability.^33,34,85^

Of particular interest, we found a congruent lateralization between increased temporal lobe tau pathology and mesial temporal epileptiform activity. Based on histopathological studies, AD was thought to be a symmetric disease. However, *in vivo* PET imaging has documented asymmetry in tau pathology in AD that is associated with more severe tau burden, earlier age of onset and faster cognitive decline.^86,87^ Murine studies demonstrated a role of neuronal activity in the release and trans-neuronal and trans-synaptic spread of tau.^5,6,88^ In a recent study amyloid-β induced hyperactivation of the mesial temporal lobe on functional MRI was associated with the rate of tau accumulation in the entorhinal cortex, but not with tau accumulation in regions compromising Braak III/IV.^10^ The significant asymmetry of temporal lobe tau pathology towards the most epileptic mesial temporal lobe further supports the hypothesis that neuronal hyperexcitability influences tau pathology, however no causal relationship can be established. Different from the observations of Giorgio *et al.*,^10^ in our sample, the lateral temporal lobe was involved as well. This might be explained by the fact that we focused on patients with a prodromal to moderate AD stage, wherein a more extended neocortical spread of tau is inherent.^2^ However, we might speculate that epileptiform activity, as a more expressed manifestation of the disrupted excitatory-inhibitory balance, drives the spread of tau, which follows a pattern of functional connectivity, beyond the entorhinal cortex.^89,90^ Longitudinal studies with tau-PET in AD patients with and without epileptiform activity could provide more insight in the causality between neuronal hyperexcitability and tau, and whether targeting neuronal hyperexcitability in the early disease stage can effect tau pathology.

Our findings have implications for future work. First, the gap between intracranial- and scalp EEG emphasizes the emerging need for other biomarkers to identify epileptiform activity. Different methods have been described to detect epileptiform activity on scalp EEG. For instance, quantitative neuronal synchrony abnormalities^91^ had the capacity to distinguish AD patients with SEA and those without. At present, these methods still need validation on combined scalp-intracranial EEG recordings. Artificial intelligence algorithms^92^ were also able to detect mesial temporal spikes, which were not discernible upon visual assessment of scalp EEG. Increasing the sensitivity of scalp EEG by integrating different methods might have additional value in clinical studies. Second, the amount and patterns of epileptiform activity present on intracranial electrodes reflect the spectrum of neuronal hyperexcitability. Longitudinal assessment needs to elucidate to which extent epileptiform activity contributes to the rate of cognitive decline. Subsequently, considering that ASM are currently investigated as potential treatment for neuronal hyperexcitability, the limited sensitivity of scalp EEG in detecting temporal lobe epileptiform activity might interfere not only with patient stratification but also with evaluating treatment effects of ASM. In the study of Bakker *et al*.^17^ a low dose levetiracetam (125 mg twice daily) reduced neuronal hyperexcitability and had a beneficial effect on cognitive functioning in patients with amnestic MCI. In the study of the effect of levetiracetam on cognition in patients with AD,^16^ the same dose of levetiracetam had only a small benefit in spatial navigating and executive functioning in AD patients with SEA on scalp EEG compared to patients without SEA. Longitudinal assessment will need to monitor the effect of ASM on IEDs and elucidate if certain patients might require a more aggressive treatment with ASM. Disruption of the excitatory-inhibitory balance of the default mode network has been associated with hyperexcitability of the mesial temporal lobe and increased longitudinal mesial temporal tau accumulation.^10^ To restore the balance between excitation and inhibition neuromodulation is currently investigated as a therapeutic option in AD.^93^ A recent randomized sham-controlled trial with repetitive transcranial magnetic stimulation (rTMS) targeting the precuneus showed promising results with respect to cognitive decline.^94^ Future studies should further focus on the non-invasive detection and modulation of aberrant neuronal excitability, as well as the optimal stimulation to restore the excitation-inhibition balance.

A main strength of our study is the simultaneously performed multi-day scalp-FO EEG/PSG recordings. This set-up allows to account for day-to-day fluctuations and to reliably document epileptiform activity and REM sleep scoring, which can be challenging using scalp EEG alone. The combination of intracranial EEG with ^18^F-MK6240 PET enabled *in vivo* documentation of both epileptiform activity and tau pathology. Another strength is that we included AD patients from the prodromal to the moderate dementia stage. This gave us opportunity to study neuronal hyperexcitability through the disease spectrum. Yet, some weaknesses were also present. First, it should be noted that only patients from our initial study cohort who had SEA on scalp EEG^45^ or with a suspicion of seizures, were recruited. Therefore we cannot extrapolate our findings to the global and heterogeneous AD population. Direct intracranial recordings or other reliable methods are needed to study to which extent AD patients are affected. Second, inherent to the small sample size we did not control for the use of medication, age and other clinical parameters. A third limitation is that Apolipoprotein ɛ status was not available. The apolipoprotein ɛ4 allele is associated with an increased risk of late onset epilepsy^95^ and sleep apnoea^96^ and affects amyloid-β and tau pathology in AD.^97^ Fourth, PSG analysis was limited to the macrostructure of sleep, and associations between microstructural sleep properties and epileptiform activity were not studied.

## Conclusion

In summary, the lack of clear clinical signs and insensitivity of scalp EEG to detect mesial temporal epileptic activity in AD patients with subclinical seizures or epileptiform activity suggests an underestimated ‘prevalence’ and ‘frequency’ of epileptiform activity from early in the AD course. Neuronal hyperexcitability in this AD subgroup encompasses a broad range with individual IEDs, rhythmic and periodic patterns, BIRDs and seizures. Epileptic activity on intracranial electrodes was modulated by sleep and its congruent lateralized character with asymmetric temporal lobe tau distribution suggests a complex multi-directional interaction. More research is necessary to further elucidate the exact mechanisms and to which extent epileptiform activity affects the AD pathogenesis, also in patients without subclinical seizures or epileptiform activity on scalp EEG. We incite a framework for longitudinal studies with a focus on an integrated approach to further explore the role of neuronal hyperexcitability, sleep alterations and tau pathology in the AD pathogenesis.

## Supplementary Material

awae231_Supplementary_Data

## Data Availability

All data are available upon request.

## References

[1] Jack CR Jr, Knopman DS, Jagust WJ, et al Tracking pathophysiological processes in Alzheimer’s disease: An updated hypothetical model of dynamic biomarkers. Lancet Neurol. 2013;12:207–216. 23332364 10.1016/S1474-4422(12)70291-0 PMC3622225

[2] Masters CL, Bateman R, Blennow K, et al Alzheimer’s disease. Nat Rev Dis Primers. 2015;1:15056. 27188934 10.1038/nrdp.2015.56

[3] Palop JJ, Mucke L. Network abnormalities and interneuron dysfunction in Alzheimer disease. Nat Rev Neurol. 2016;17:777–792. 10.1038/nrn.2016.141 PMC8162106 27829687

[4] Romoli M, Sen A, Parnetti L, Calabresi P, Costa C. Amyloid-β: A potential link between epilepsy and cognitive decline. Nat Rev Neurol. 2021;17:469–485. 34117482 10.1038/s41582-021-00505-9

[5] Wu JW, Hussaini SA, Bastille IM, et al Neuronal activity enhances tau propagation and tau pathology in vivo. Nat Neurosci. 2016;19:1085–1092. 27322420 10.1038/nn.4328 PMC4961585

[6] Rodriguez GA, Barrett GM, Duff KE, Hussaini SA. Chemogenetic attenuation of neuronal activity in the entorhinal cortex reduces Aβ and tau pathology in the hippocampus. PLoS Biol. 2020;18:e3000851. 32822389 10.1371/journal.pbio.3000851 PMC7467290

[7] Busche MA, Konnerth A. Impairments of neural circuit function in Alzheimer’s disease. Philos Trans R Soc Lond B Biol Sci. 2016;371:20150429. 27377723 10.1098/rstb.2015.0429 PMC4938029

[8] Busche MA, Chen X, Henning HA, et al Critical role of soluble amyloid-β for early hippocampal hyperactivity in a mouse model of Alzheimer’s disease. Proc Natl Acad Sci U S A. 2012;109:8740–8745. 22592800 10.1073/pnas.1206171109 PMC3365221

[9] Roberson ED, Scearce-Levie K, Palop JJ, et al Reducing endogenous tau ameliorates amyloid beta-induced deficits in an Alzheimer’s disease mouse model. Science. 2007;316:750–754. 17478722 10.1126/science.1141736

[10] Giorgio J, Adams JN, Maass A, Jagust WJ, Breakspear M. Amyloid induced hyperexcitability in default mode network drives medial temporal hyperactivity and early tau accumulation. Neuron. 2023;112:676–686.e4. 38096815 10.1016/j.neuron.2023.11.014 PMC10922797

[11] Pontecorvo MJ, Devous MD, Navitsky M, et al Relationships between flortaucipir PET tau binding and amyloid burden, clinical diagnosis, age and cognition. Brain. 2017;140:748–763. 28077397 10.1093/brain/aww334 PMC5382945

[12] Tai XY, Koepp M, Duncan JS, et al Hyperphosphorylated tau in patients with refractory epilepsy correlates with cognitive decline: A study of temporal lobe resections. Brain. 2016;139(Pt 9):2441–2455. 27497924 10.1093/brain/aww187 PMC5926008

[13] Bejanin A, Schonhaut DR, La Joie R, et al Tau pathology and neurodegeneration contribute to cognitive impairment in Alzheimer’s disease. Brain. 2017;140:3286–3300. 29053874 10.1093/brain/awx243 PMC5841139

[14] Horvath AA, Papp A, Zsuffa J, et al Subclinical epileptiform activity accelerates the progression of Alzheimer’s disease: A long-term EEG study. Clin Neurophysiol. 2021;132:1982–1989. 34034963 10.1016/j.clinph.2021.03.050

[15] Vossel KA, Ranasinghe KG, Beagle AJ, et al Incidence and impact of subclinical epileptiform activity in Alzheimer’s disease. Ann Neurol. 2016;80:858–870. 27696483 10.1002/ana.24794 PMC5177487

[16] Vossel KA, Ranasinghe KG, Beagle AJ, et al Effect of levetiracetam on cognition in patients with Alzheimer disease with and without epileptiform activity: A randomized clinical trial. JAMA Neurol. 2021;78:1345–1354. 34570177 10.1001/jamaneurol.2021.3310 PMC8477304

[17] Bakker A, Krauss GL, Albert MS, et al Reduction of hippocampal hyperactivity improves cognition in amnestic mild cognitive impairment. Neuron. 2012;74:467–474. 22578498 10.1016/j.neuron.2012.03.023 PMC3351697

[18] Ju YE, Lucey BP, Holtzman DM. Sleep and Alzheimer disease pathology—A bidirectional relationship. Nat Rev Neurol. 2014;10:115–119. 24366271 10.1038/nrneurol.2013.269 PMC3979317

[19] Falter A, Van Den Bossche MJA. How non-rapid eye movement sleep and Alzheimer pathology are linked. World J Psychiatry. 2021;11:1027–1038. 34888171 10.5498/wjp.v11.i11.1027 PMC8613758

[20] Mander BA, Winer JR, Jagust WJ, Walker MP. Sleep: A novel mechanistic pathway, biomarker, and treatment target in the pathology of Alzheimer’s disease? Trends Neurosci. 2016;39:552–566. 27325209 10.1016/j.tins.2016.05.002 PMC4967375

[21] Lucey BP, McCullough A, Landsness EC, et al Reduced non-rapid eye movement sleep is associated with tau pathology in early Alzheimer’s disease. Sci Transl Med. 2019;11:eaau6550. 30626715 10.1126/scitranslmed.aau6550 PMC6342564

[22] Lucey BP, Wisch J, Boerwinkle AH, et al Sleep and longitudinal cognitive performance in preclinical and early symptomatic Alzheimer’s disease. Brain. 2021;144:2852–2862. 34668959 10.1093/brain/awab272 PMC8536939

[23] Díaz-Román M, Pulopulos MM, Baquero M, et al Obstructive sleep apnoea and Alzheimer’s disease-related cerebrospinal fluid biomarkers in mild cognitive impairment. Sleep. 2021;44:zsaa133. 32728730 10.1093/sleep/zsaa133

[24] Liguori C, Romigi A, Nuccetelli M, et al Orexinergic system dysregulation, sleep impairment, and cognitive decline in Alzheimer disease. JAMA Neurol. 2014;71:1498–1505. 25322206 10.1001/jamaneurol.2014.2510

[25] Shokri-Kojori E, Wang GJ, Wiers CE, et al β-Amyloid accumulation in the human brain after one night of sleep deprivation. Proc Natl Acad Sci U S A. 2018;115:4483–4488. 29632177 10.1073/pnas.1721694115 PMC5924922

[26] Olsson M, Ärlig J, Hedner J, Blennow K, Zetterberg H. Sleep deprivation and cerebrospinal fluid biomarkers for Alzheimer’s disease. Sleep. 2018;41:zsy025. 10.1093/sleep/zsy025 29425372

[27] Ju YS, Ooms SJ, Sutphen C, et al Slow wave sleep disruption increases cerebrospinal fluid amyloid-beta levels. Brain. 2017;140:2104–2111. 28899014 10.1093/brain/awx148 PMC5790144

[28] Winer JR, Mander BA, Kumar S, et al Sleep disturbance forecasts β-amyloid accumulation across subsequent years. Curr Biol. 2020;30:4291–4298.e3. 32888482 10.1016/j.cub.2020.08.017 PMC7642104

[29] Ooms S, Overeem S, Besse K, et al Effect of 1 night of total sleep deprivation on cerebrospinal fluid β-amyloid 42 in healthy middle-aged men. JAMA Neurol. 2014;71:971–977. 24887018 10.1001/jamaneurol.2014.1173

[30] Winer JR, Mander BA, Helfrich RF, et al Sleep as a potential biomarker of tau and β-amyloid burden in the human brain. J Neurosci. 2019;39:6315–6324. 31209175 10.1523/JNEUROSCI.0503-19.2019 PMC6687908

[31] Varga AW, Wohlleber ME, Gimenez S, et al Reduced slow-wave sleep is associated with high cerebrospinal fluid Aβ42 levels in cognitively normal elderly. Sleep. 2016;39:2041–2048. 27568802 10.5665/sleep.6240 PMC5070758

[32] Mander BA, Marks SM, Vogel JW, et al β-amyloid disrupts human NREM slow waves and related hippocampus-dependent memory consolidation. Nat Neurosci. 2015;18:1051–1057. 26030850 10.1038/nn.4035 PMC4482795

[33] Bu XL, Liu YH, Wang QH, et al Serum amyloid-beta levels are increased in patients with obstructive sleep apnoea syndrome. Sci Rep. 2015;5:13917. 26351108 10.1038/srep13917 PMC4563592

[34] Ju YS, Finn MB, Sutphen CL, et al Obstructive sleep apnoea decreases central nervous system-derived proteins in the cerebrospinal fluid. Ann Neurol. 2016;80:154–159. 27129429 10.1002/ana.24672 PMC5120585

[35] Romanella SM, Roe D, Tatti E, et al The sleep side of aging and Alzheimer’s disease. Sleep Med. 2021;77:209–225. 32912799 10.1016/j.sleep.2020.05.029 PMC8364256

[36] Zhang Y, Ren R, Yang L, et al Sleep in Alzheimer’s disease: A systematic review and meta-analysis of polysomnographic findings. Transl Psychiatry. 2022;12:136. 35365609 10.1038/s41398-022-01897-y PMC8976015

[37] Bubu OM, Andrade AG, Umasabor-Bubu OQ, et al Obstructive sleep apnoea, cognition and Alzheimer’s disease: A systematic review integrating three decades of multidisciplinary research. Sleep Med Rev. 2020;50:101250. 31881487 10.1016/j.smrv.2019.101250 PMC7593825

[38] Liu S, Pan J, Tang K, et al Sleep spindles, K-complexes, limb movements and sleep stage proportions may be biomarkers for amnestic mild cognitive impairment and Alzheimer’s disease. Sleep Breath. 2020;24:637–651. 31786748 10.1007/s11325-019-01970-9

[39] Westerberg CE, Mander BA, Florczak SM, et al Concurrent impairments in sleep and memory in amnestic mild cognitive impairment. J Int Neuropsychol Soc. 2012;18:490–500. 22300710 10.1017/S135561771200001X PMC3468412

[40] Grimaldi D, Papalambros NA, Zee PC, Malkani RG. Neurostimulation techniques to enhance sleep and improve cognition in aging. Neurobiol Dis. 2020;141:104865. 32251840 10.1016/j.nbd.2020.104865

[41] Nobili L, Frauscher B, Eriksson S, et al Sleep and epilepsy: A snapshot of knowledge and future research lines. J Sleep Res. 2022;31:e13622. 35487880 10.1111/jsr.13622 PMC9540671

[42] Frauscher B, von Ellenrieder N, Ferrari-Marinho T, et al Facilitation of epileptic activity during sleep is mediated by high amplitude slow waves. Brain. 2015;138(Pt 6):1629–1641. 25792528 10.1093/brain/awv073 PMC4614129

[43] Peter-Derex L, Klimes P, Latreille V, et al Sleep disruption in epilepsy: Ictal and interictal epileptic activity matter. Ann Neurol. 2020;88:907–920. 32833279 10.1002/ana.25884

[44] Tabuchi M, Lone SR, Liu S, Liu Q, Zhang J, Spira AP, Wu MN. Sleep interacts with Aβ to modulate intrinsic neuronal excitability. Curr Biol. 2015;25:702–712. 25754641 10.1016/j.cub.2015.01.016 PMC4366315

[45] Devulder A, Macea J, Kalkanis A, et al Subclinical epileptiform activity and sleep disturbances in Alzheimer’s disease. Brain Behav. 2023;13:e3306. 37950422 10.1002/brb3.3306 PMC10726840

[46] Ciliento R, Gjini K, Dabbs K, et al Prevalence and localization of nocturnal epileptiform discharges in mild cognitive impairment. Brain Commun. 2023;5:fcad302. 37965047 10.1093/braincomms/fcad302 PMC10642616

[47] Lam AD, Sarkis RA, Pellerin KR, et al Association of epileptiform abnormalities and seizures in Alzheimer disease. Neurology. 2020;95:e2259–e2270. 32764101 10.1212/WNL.0000000000010612 PMC7713786

[48] Lieb JP, Walsh GO, Babb TL, Walter RD, Crandall PH. A comparison of EEG seizure patterns recorded with surface and depth electrodes in patients with temporal lobe epilepsy. Epilepsia. 1976;17:137–160. 947745 10.1111/j.1528-1157.1976.tb03392.x

[49] Lam AD, Deck G, Goldman A, et al Silent hippocampal seizures and spikes identified by foramen ovale electrodes in Alzheimer’s disease. Nat Med. 2017;23:678–680. 28459436 10.1038/nm.4330 PMC5461182

[50] Velasco TR, Sakamoto AC, Alexandre V Jr, et al Foramen ovale electrodes can identify a focal seizure onset when surface EEG fails in mesial temporal lobe epilepsy. Epilepsia. 2006;47:1300–1307. 16922874 10.1111/j.1528-1167.2006.00547.x

[51] Sheth SA, Aronson JP, Shafi MM, et al Utility of foramen ovale electrodes in mesial temporal lobe epilepsy. Epilepsia. 2014;55:713–724. 24605889 10.1111/epi.12571

[52] McKhann GM, Knopman DS, Chertkow H, et al The diagnosis of dementia due to Alzheimer’s disease: Recommendations from the National Institute on Aging-Alzheimer’s Association workgroups on diagnostic guidelines for Alzheimer’s disease. Alzheimers Dement. 2011;7:263–269. 21514250 10.1016/j.jalz.2011.03.005 PMC3312024

[53] Albert MS, DeKosky ST, Dickson D, et al The diagnosis of mild cognitive impairment due to Alzheimer’s disease: Recommendations from the National Institute on Aging-Alzheimer’s Association workgroups on diagnostic guidelines for Alzheimer’s disease. Alzheimers Dement. 2011;7:270–279. 21514249 10.1016/j.jalz.2011.03.008 PMC3312027

[54] Jack CR, Bennett DA, Blennow K, et al NIA-AA research framework: Toward a biological definition of Alzheimer’s disease. Alzheimers Dement. 2018;14:535–562. 29653606 10.1016/j.jalz.2018.02.018 PMC5958625

[55] Reutens DC, Howell RA, Gebert KE, Berkovic SF. Validation of a questionnaire for clinical seizure diagnosis. Epilepsia. 1992;33:1065–1071. 1464265 10.1111/j.1528-1157.1992.tb01760.x

[56] Seeck M, Koessler L, Bast T, et al The standardized EEG electrode array of the IFCN. J Clin Neurophysiol. 2017;128:2070–2077. 10.1016/j.clinph.2017.06.254 28778476

[57] Hirsch LJ, Fong MWK, Leitinger M, et al American Clinical Neurophysiology Society’s standardized critical care EEG terminology: 2021 version. J Clin Neurophysiol. 2021;38:1–29. 33475321 10.1097/WNP.0000000000000806 PMC8135051

[58] Kural MA, Duez L, Sejer Hansen V, et al Criteria for defining interictal epileptiform discharges in EEG: A clinical validation study. Neurology. 2020;94:e2139–e2147. 32321764 10.1212/WNL.0000000000009439 PMC7526669

[59] Scheuer ML, Bagic A, Wilson SB. Spike detection: Inter-reader agreement and a statistical Turing test on a large data set. Clin Neurophysiol. 2017;128:243–250. 27913148 10.1016/j.clinph.2016.11.005

[60] Reus EEM, Cox FME, Van Dijk JG, Visser GH. Automated spike detection: Which software package? Seizure. 2022;95:33–37. 34974231 10.1016/j.seizure.2021.12.012

[61] Berry RB, Quan SF, Abreu AR, et al The AASM manual for the scoring of sleep and associated events: Rules, terminology and technical specifications. Version 2.6. American Academy of Sleep Medicine. American Academy of Sleep Medicine; 2020.

[62] Mertens N, Michiels L, Vanderlinden G, et al Impact of meningeal uptake and partial volume correction techniques on [(18)F]MK-6240 binding in aMCI patients and healthy controls. J Cereb Blood Flow Metab. 2022;42:1236–1246. 35062837 10.1177/0271678X221076023 PMC9207493

[63] Reeves AL, Nollet KE, Klass DW, Sharbrough FW, So EL. The ictal bradycardia syndrome. Epilepsia. 1996;37:983–987. 8822697 10.1111/j.1528-1157.1996.tb00536.x

[64] Tinuper P . Ictal bradycardia in partial epileptic seizures: Autonomic investigation in three cases and literature review. Brain. 2001;124:2361–2371. 11701591 10.1093/brain/124.12.2361

[65] Ungar A, Mussi C, Ceccofiglio A, et al Etiology of syncope and unexplained falls in elderly adults with dementia: Syncope and dementia (SYD) study. J Am Geriatr Soc. 2016;64:1567–1573. 27351866 10.1111/jgs.14225

[66] Vossel KA, Beagle AJ, Rabinovici GD, et al Seizures and epileptiform activity in the early stages of Alzheimer disease. JAMA Neurol. 2013;70:1158–1166. 23835471 10.1001/jamaneurol.2013.136 PMC4013391

[67] Yoo JY, Marcuse LV, Fields MC, et al Brief potentially ictal rhythmic discharges [B(I)RDs] in noncritically ill adults. J Clin Neurophysiol. 2017;34:222–229. 28463933 10.1097/WNP.0000000000000357

[68] Rodriguez Ruiz A, Vlachy J, Lee JW, et al Association of periodic and rhythmic electroencephalographic patterns with seizures in critically ill patients. JAMA Neurol. 2017;74:181. 27992625 10.1001/jamaneurol.2016.4990

[69] Di Gennaro G, Quarato PP, Onorati P, et al Localizing significance of temporal intermittent rhythmic delta activity (TIRDA) in drug-resistant focal epilepsy. Clin Neurophysiol. 2003;114:70–78. 12495766 10.1016/s1388-2457(02)00332-2

[70] Gambardella A, Gotman J, Cendes F, Andermann F. Focal intermittent delta activity in patients with mesiotemporal atrophy: A reliable marker of the epileptogenic focus. Epilepsia. 1995;36:122–129. 7821268 10.1111/j.1528-1157.1995.tb00970.x

[71] Diekelmann S, Born J. The memory function of sleep. Nat Rev Neurosci. 2010;11:114–126. 20046194 10.1038/nrn2762

[72] Mander BA . Local sleep and Alzheimer’s disease pathophysiology. Front Neurosci. 2020;14:525970. 33071726 10.3389/fnins.2020.525970 PMC7538792

[73] Kleen JK, Scott RC, Holmes GL, et al Hippocampal interictal epileptiform activity disrupts cognition in humans. Neurology. 2013;81:18–24. 23685931 10.1212/WNL.0b013e318297ee50 PMC3770206

[74] Lambert I, Tramoni-Negre E, Lagarde S, et al Hippocampal interictal spikes during sleep impact long-term memory consolidation. Ann Neurol. 2020;87:976–987. 32279329 10.1002/ana.25744

[75] Malow A, Bowes RJ, Ross D. Relationship of temporal lobe seizures to sleep and arousal: A combined scalp-intracranial electrode study. Sleep. 2000;23:231–234. 10737340

[76] Lam AD, Zepeda R, Cole AJ, Cash SS. Widespread changes in network activity allow non-invasive detection of mesial temporal lobe seizures. Brain. 2016;139(Pt 10):2679–2693. 27474219 10.1093/brain/aww198 PMC5035820

[77] Li W, Ma L, Yang G, Gan W-B. REM sleep selectively prunes and maintains new synapses in development and learning. Nat Neurosci. 2017;20:427–437. 28092659 10.1038/nn.4479 PMC5535798

[78] D’Atri A, Scarpelli S, Gorgoni M, et al EEG alterations during wake and sleep in mild cognitive impairment and Alzheimer’s disease. iScience. 2021;24:102386. 33981973 10.1016/j.isci.2021.102386 PMC8086022

[79] Lin Z, Si Q, Xiaoyi Z. Obstructive sleep apnoea in patients with epilepsy: A meta-analysis. Sleep Breath. 2017;21:263–270. 27473532 10.1007/s11325-016-1391-3

[80] Malow BA, Weatherwax KJ, Chervin RD, et al Identification and treatment of obstructive sleep apnoea in adults and children with epilepsy: A prospective pilot study. Sleep Med. 2003;4:509–515. 14607344 10.1016/j.sleep.2003.06.004

[81] Vendrame M, Auerbach S, Loddenkemper T, Kothare S, Montouris G. Effect of continuous positive airway pressure treatment on seizure control in patients with obstructive sleep apnoea and epilepsy. Epilepsia. 2011;52:e168–e171. 21849000 10.1111/j.1528-1167.2011.03214.x

[82] Pornsriniyom D, Shinlapawittayatorn K, Fong J, Andrews ND, Foldvary-Schaefer N. Continuous positive airway pressure therapy for obstructive sleep apnoea reduces interictal epileptiform discharges in adults with epilepsy. Epilepsy Behav. 2014;37:171–174. 25042599 10.1016/j.yebeh.2014.06.025

[83] Oliveira AJ, Zamagni M, Dolso P, Bassetti MA, Gigli GL. Respiratory disorders during sleep in patients with epilepsy: Effect of ventilatory therapy on EEG interictal epileptiform discharges. Clin Neurophysiol. 2000;111(Suppl 2):S141–S145. 10996568 10.1016/s1388-2457(00)00415-6

[84] Pornsriniyom D, Kim H, Bena J, et al Effect of positive airway pressure therapy on seizure control in patients with epilepsy and obstructive sleep apnoea. Epilepsy Behav. 2014;37:270–275. 25117208 10.1016/j.yebeh.2014.07.005

[85] Shiota S, Takekawa H, Matsumoto S-E, et al Chronic intermittent hypoxia/reoxygenation facilitate amyloid-β generation in mice. J Alzheimers Dis. 2013;37:325–333. 23948880 10.3233/JAD-130419

[86] Lu J, Zhang Z, Wu P, et al The heterogeneity of asymmetric tau distribution is associated with an early age at onset and poor prognosis in Alzheimer’s disease. Neuroimage Clin. 2023;38:103416. 37137254 10.1016/j.nicl.2023.103416 PMC10176076

[87] Vogel JW, Young AL, Oxtoby NP, et al Four distinct trajectories of tau deposition identified in Alzheimer’s disease. Nat Med. 2021;27:871–881. 33927414 10.1038/s41591-021-01309-6 PMC8686688

[88] Schultz MK Jr, Gentzel R, Usenovic M, et al Pharmacogenetic neuronal stimulation increases human tau pathology and trans-synaptic spread of tau to distal brain regions in mice. Neurobiol Dis. 2018;118:161–176. 30049665 10.1016/j.nbd.2018.07.003

[89] Vogel JW, Iturria-Medina Y, Strandberg OT, et al Spread of pathological tau proteins through communicating neurons in human Alzheimer’s disease. Nat Commun. 2020;11:2612. 32457389 10.1038/s41467-020-15701-2 PMC7251068

[90] Franzmeier N, Neitzel J, Rubinski A, et al Functional brain architecture is associated with the rate of tau accumulation in Alzheimer’s disease. Nat Commun. 2020;11:347. 31953405 10.1038/s41467-019-14159-1 PMC6969065

[91] Ranasinghe KG, Kudo K, Hinkley L, et al Neuronal synchrony abnormalities associated with subclinical epileptiform activity in early-onset Alzheimer’s disease. Brain. 2022;145:744–753. 34919638 10.1093/brain/awab442 PMC9630715

[92] Abou Jaoude M, Jacobs CS, Sarkis RA, et al Noninvasive detection of hippocampal epileptiform activity on scalp electroencephalogram. JAMA Neurol. 2022;79:614–622. 35499837 10.1001/jamaneurol.2022.0888 PMC9062772

[93] Millet B, Mouchabac S, Robert G, et al Transcranial magnetic stimulation (rTMS) on the precuneus in Alzheimer’s disease: A literature review. Brain Sci. 2023;13:1332. 37759933 10.3390/brainsci13091332 PMC10526400

[94] Koch G, Casula EP, Bonnì S, et al Precuneus magnetic stimulation for Alzheimer’s disease: A randomized, sham-controlled trial. Brain. 2022;145:3776–3786. 36281767 10.1093/brain/awac285 PMC9679166

[95] Johnson EL, Krauss GL, Lee AK, et al Association between midlife risk factors and late-onset epilepsy. JAMA Neurol. 2018;75:1375–1382. 30039175 10.1001/jamaneurol.2018.1935 PMC6248112

[96] Kadotani H, Kadotani T, Young T, et al Association between apolipoprotein E ∊4 and sleep-disordered breathing in adults. JAMA. 2001;285:2888. 11401610 10.1001/jama.285.22.2888

[97] Koutsodendris N, Nelson MR, Rao A, Huang Y. Apolipoprotein E and Alzheimer’s disease: Findings, hypotheses, and potential mechanisms. Annu Rev Pathol Mech Dis. 2022;17:73–99. 10.1146/annurev-pathmechdis-030421-112756 34460318

