# Supplementary material for: Epileptic activity on foramen ovale electrodes is associated with sleep and tau pathology in Alzheimer’s disease
Source: Brain. 2024 Jul 11;148(2):506–20. doi: 10.1093/brain/awae231 (PMC11788210; doi:10.1093/brain/awae231)
Supplement: awae231_Supplementary_Data [file awae231_supplementary_data.pdf]

SUPPLEMENT

Content

1. Supplementary Methods 2

1.1 Biomarker assessment 2

1.2 EEG review process 2

2. Supplementary Tables 3

2.1 Supplementary Table 1 Biomarker and imaging data 3

2.2 Supplementary Table 2 Linear Mixed effects models of the relationship between epileptiform activity on FO electrodes and PSG parameters 5

2.3 Supplementary Table 3 Polysomnography parameters for each patient over different nights* 6

3. Supplementary Figures 8

3.1 Supplementary Figure 1 8

3.2 Supplementary Figure 2 9

3.3 Supplementary Figure 3 10

3.4 Supplementary Figure 4 11

4. References 13

1. Supplementary Methods

1.1 Biomarker assessment

Biomarkers in the cerebrospinal fluid (CSF) for Alzheimer's disease (AD) were assessed with the Enzyme Linked Immune Sorbent Assay (ELISA) method from Innogenetics (Zwijnaarde, Belgium) for the first patient and with the ELISA method from Euroimmun (Lübeck, Germany) for the remaining patients.

1.2 EEG review process

We used the Persyst 14 Spike Detector Software (Persyst, CA, USA) to detect interictal epileptiform discharges (IEDs) on both foramen ovale (FO) electrodes and scalp EEG, from 'lights off' until 'lights on'. The filter was set at 'low sensitivity' to minimize the false positive rate.[1,2] All IEDs annotated by the Persyst 14 software, were next visually reviewed by first reader (A.D.). IEDs, marked by the Persyst 14 software, that not fulfilled the International Federation for Clinical Neurophysiology (IFCN) criteria[3], were discarded. In case of ambiguity, the IEDs were discussed with second reader (W.V.P.). Next, for both scalp EEG and FO electrodes, the remaining IEDs were quantified per hour. Small sharp spikes of sleep and wicket spikes were not included as epileptiform activity. In addition, first reader (A.D.) visually reviewed all scalp and FO EEG recordings for rhythmic and periodic patterns according to the American Clinical Neurophysiology Society (ACNS) criteria.[4] These annotations were next discussed with the second reader (W.V.P.). In case of disagreement, consensus was reached after open discussion. Seizures, lateralized rhythmic delta activity (LRDA), periods of lateralized periodic discharges (LPDs) and brief potentially ictal rhythmic discharges (BIRDs) were annotated and the prevalence, frequency and duration of these patterns for each overnight recording was described.

2. Supplementary Tables

2.1 Supplementary Table 1 Biomarker and imaging data

Subject	Disease stage	Amyloid PET	CSF Biomarkers	^{18}F- FDG PET	Brain MRI
1	Moderate AD	NA	Aβ 1-42 = 332<br>t-tau = 770<br>p-tau = 107.8<br>Ratio Aβ 1-42/t-tau = 0.431 [a]	Hypometabolism temporoparietal R > L, precuneus and frontal L	Generalized atrophy, parietal and temporal predominance. Prominent mesial temporal atrophy R > L
2	Mild AD	NA	Aβ 1-42 = 361<br>Ratio 1-42/1-40 = 0.051<br>t-tau = 71<br>p-tau = 110<br>Ratio Aβ 1-42/t-tau = 0.505 [b]	NA	Generalized atrophy. Prominent mesial temporal atrophy. Moderate vascular white matter lesions. Gliosis frontoparietal right.
3	Prodromal AD	NA	Aβ 1-42 = 667<br>Ratio 1-42/1-40 = 0.075<br>t-tau = 674<br>Ratio Aβ 1-42/t-tau = 0.990 [b]	Hypometabolism temporoparietal bilateral, frontal bilateral, precuneus and posterior cingulate.	Mild generalized atrophy. Prominent parietal atrophy. Mild mesial temporal atrophy L > R. Mild vascular lesions (Fazekas 1).
4	Prodromal AD	NA	Aβ 1-42 = 370<br>Ratio 1-42/1-40 = 0.061<br>t-tau = 389<br>Ratio Aβ 1-42/t-tau = 0.951 [b]	Mild hypometabolism posterior cingulate.	Generalized atrophy, parieto-occipital predominance. Prominent mesial temporal atrophy. Prominent white matter lesions.
5	Prodromal AD	NA	Aβ 1-42 = 439<br>Ratio 1-42/1-40 = 0.046<br>t-tau = 1173<br>Ratio Aβ 1-42/t-tau = 0.374 [b]	Mild hypometabolism temporoparietal, bilateral precuneus and posterior cingulate.	Mild generalized atrophy. Mild parietal atrophy. Mild mesial temporal atrophy.

Abbreviations: Aβ 1-40 = Amyloid-beta amyloid-β peptide ending in amino acid residue 40; Aβ 1-42 = Amyloid-beta amyloid-β peptide ending in amino acid residue 42; L = Left; NA = Not available; p-tau

= Tau phosphorylated at threonine 181; R = Right; t-tau = Total tau; ^{18}F-FDG = [^{18}F]-fluoro-2-deoxyglucose.

[a] Values supporting a diagnosis of Alzheimer's disease are Aβ 1-42 < 500 pg/mL, t-tau > 367 pg/mL, p-tau > 80 pg/mL.

[b] Values supporting a diagnosis of Alzheimer's disease are ratio Aβ 1-42/ Aβ 1-40 < 0.096, t-tau > 545 pg/mL.

2.2 Supplementary Table 2 Linear Mixed effects models of the relationship between epileptiform activity on FO electrodes and PSG parameters

**Outcome**	**Variable**	**Estimate (β)**	**95% CI**	***P-value***	***Adj P-value***
Log IEDs FO TST/h	Arousal Index	0.027	[-0.039 – 0.093]	0.317	0.317
Log IEDs FO TST/h	AHI on TST	0.004	[-0.054 – 0.062]	0.846	1.000
Log IEDs FO TST/h	ODI on TST	0.006	[-0.049 – 0.062]	0.764	1.000
Log IEDs FO NREM/h	AHI NREM	0.009	[-0.043 – 0.060]	0.666	1.000
Log IEDs FO REM/h	AHI REM	-0.005	[-0.040 – 0.031]	0.777	1.000
Log IEDs FO TST/h	N1%	0.018	[-0.089 – 0.125]	0.668	1.000
Log IEDs FO TST/h	N2%	0.010	[-0.018 – 0.038]	0.442	1.000
Log IEDs FO TST/h	SWS%	< 0.001	[-0.030 – 0.030]	0.988	1.000
Log IEDs FO TST/h	REM%	-0.015	[-0.053 – 0.023]	0.415	1.000

Abbreviations: AHI = Apnea-Hypopnea Index; FO = Foramen ovale; IEDs = Interictal epileptiform discharges; NREM = Non-REM; N1 = Non-REM1; N2 = Non-REM2; ODI = Oxygen desaturation index; SWS = Slow wave sleep; TST/h = Per hour total sleep time.

2.3 Supplementary Table 3 Polysomnography parameters for each patient over different nights*

	Patient 1	Patient 2	Patient 3	Patient 4	Patient 5
Nights recorded	3	6	3	4	4
TIB (min)	638 [638 – 661]	449 [432 – 569]	587 [536 – 588]	563 [525 – 567]	572 [483 – 608]
TST (min)	464 [338 – 562]	287 [253 – 431]	437 [198 – 441]	417 [204 – 471]	399 [352 – 448]
SE (%)	73 [51 – 88]	64 [59 – 76]	74 [37 – 75]	74 [39 – 83]	73 [61 – 80]
WASO (min)	173 [64 – 302]	120 [110 – 186]	114 [113 – 199]	99 [74 – 275]	107 [102 – 150]
Sleep latency (min)	1 [1 – 1]	19 [5 – 53]	30 [21 – 69]	36 [14 – 43]	13 [5 – 64]
REM latency (min)	160 [145 – 334]	164 [143 – 216]	64 [41 – 460]	187 [73 – 434]	112 [41 – 394]
N1 (%)	18 [5 – 26]	17 [9 – 30]	6 [2 – 8]	8 [5 – 10]	11 [6 – 22]
N2 (%)	66 [54 – 68]	42 [36 – 73]	34 [32 – 59]	54 [50 – 66]	39 [32 – 61]
SWS (%)	6 [5 – 19]	31 [4 – 42]	34 [33 – 36]	23 [0 – 38]	24 [12 – 31]
REM (%)	10 [1 – 21]	4 [0 – 14]	25 [1 – 30]	16 [6 – 23]	25 [5 – 33]
Arousal index (n/h)	30 [13 – 32]	28 [17 – 42]	9 [6 – 9]	18 [16 – 25]	34 [26 – 70]
AHI (n/h)	44 [42 49]	60 [30 – 67]	43 [23 – 54]	6 [1 – 16]	29 [25 – 73]
OAHI (n/h)	42 [35 – 44]	43 [25 – 54]	43 [21 – 52]	4 [1 – 15]	28 [24 – 73]
CAHI (n/h)	5 [2 – 7]	18 [2 – 34]	1 [0 – 2]	1 [1 – 2]	1 [0 – 1]
ODI (n/h)	45 [36 – 46]	56 [31 – 61]	46 [24 – 55]	4 [2 – 15]	31 [25 – 71]
Mean saturation (%)	94 [94 – 95]	94 [92 – 94]	90 [90 – 90]	91 [91 – 91]	90 [89 – 91]

Abbreviations: AHI = Apnea-Hypopnea Index; CAHI = Central Apnea-Hypopnea Index; N1 = Non-REM1; N2 = Non-REM2; OAHI = Obstructive Apnea-Hypopnea Index; ODI = Oxygen desaturation index; SE = Sleep efficacy; SWS = Slow wave sleep; TIB = Time in bed; TST = Total sleep time; WASO = Wake after sleep onset.

*For each parameter the median with range is shown.

3. Supplementary Figures

3.1 Supplementary Figure 1

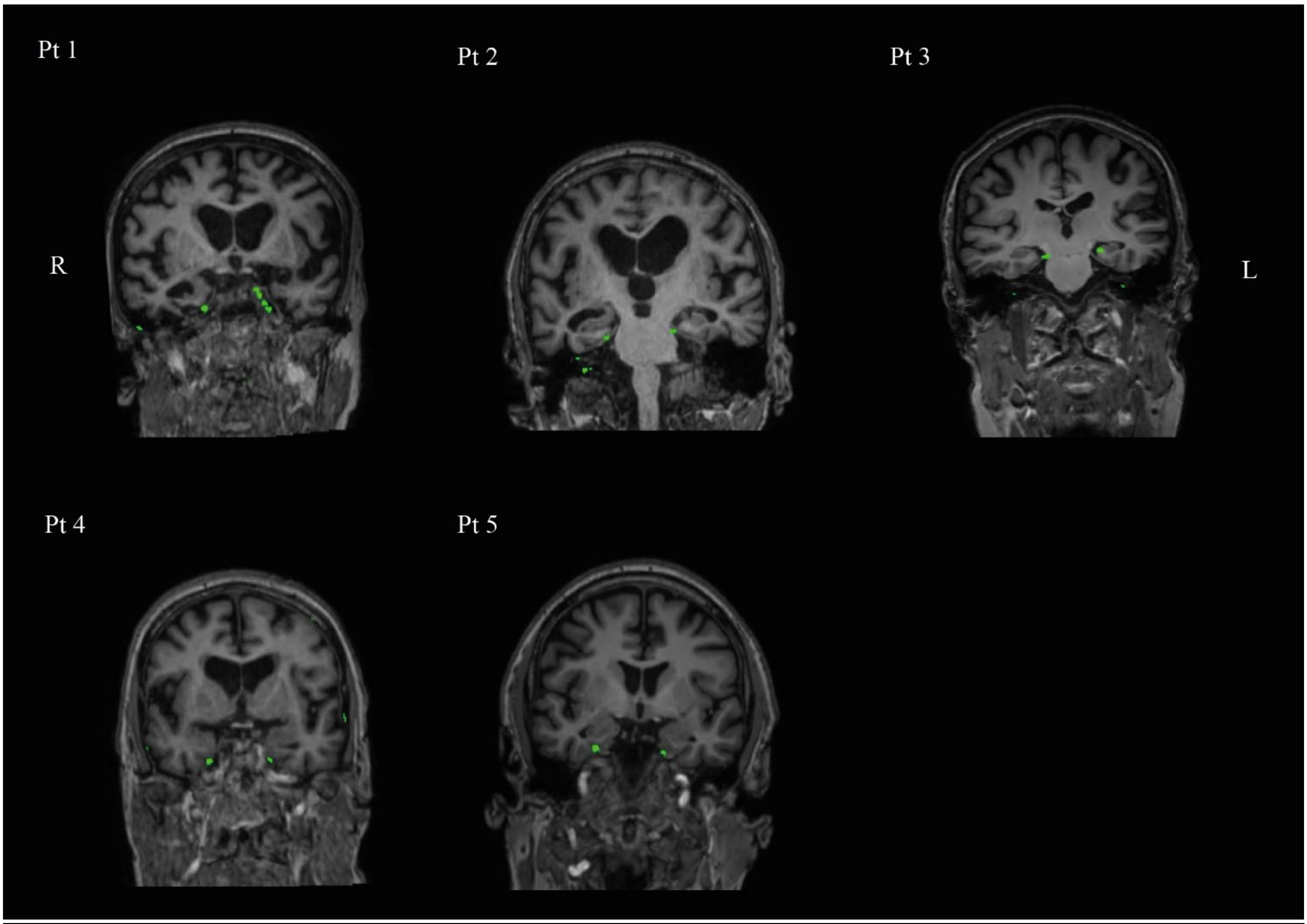

Supplementary Figure 1 FO electrode positions. Coronal post-op CT images co-registered with MRI. Green dots represent FO electrode contacts. Abbreviations: L = Left; Pt = Patient; R = Right.

3.2 Supplementary Figure 2

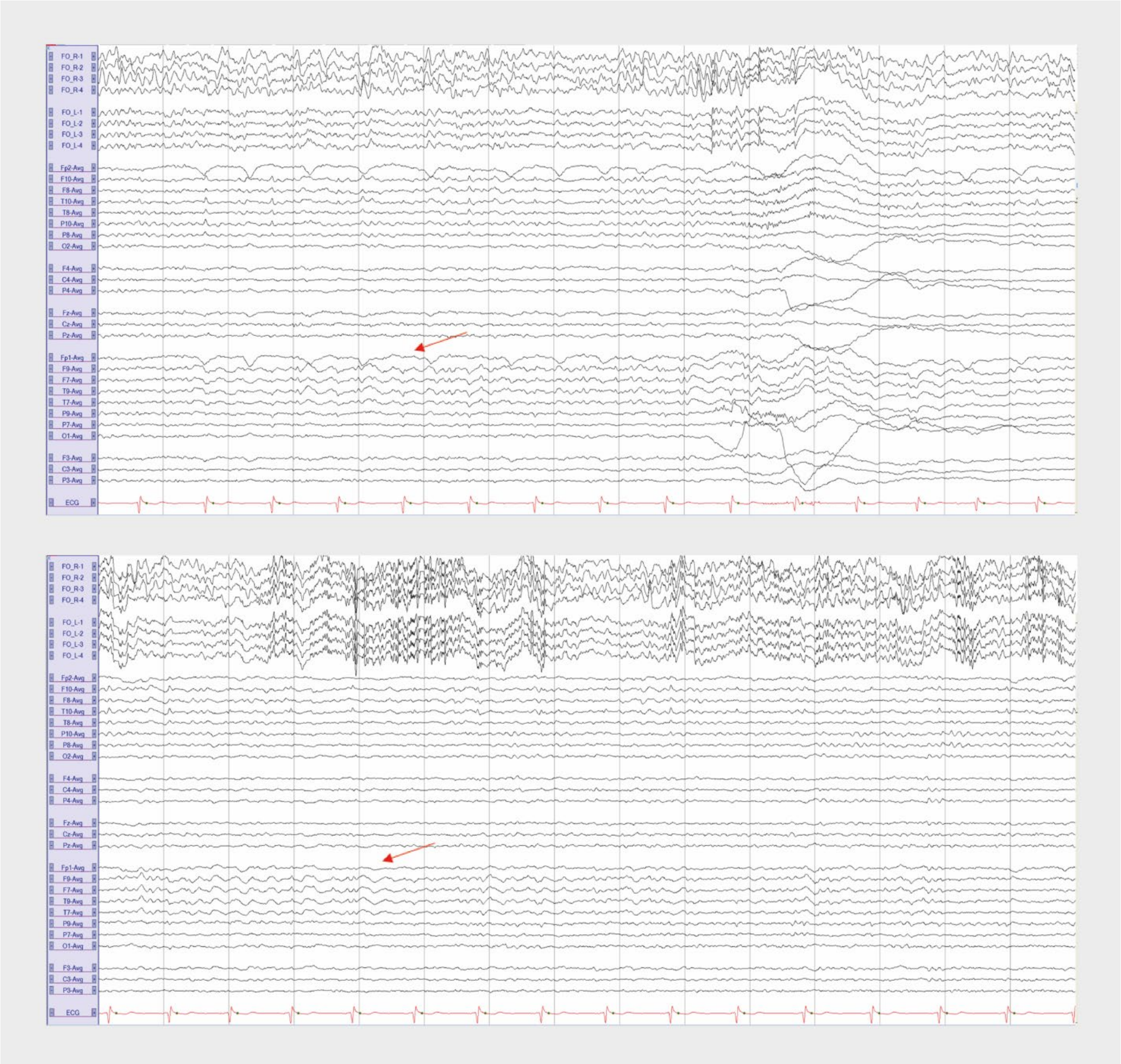

Supplementary Figure 2 Examples of temporal LRDA on scalp EEG. Lateralized rhythmic delta activity (LRDA) in the left hemisphere on scalp EEG without correlate on FO electrodes during wakefulness in patient 5. Each panels represents a 15 second epoch. Referential montage. Filters at 0.53 – 15.0 Hz. Sensitivity of 70 µV for both FO electrodes and scalp EEG.

3.3 Supplementary Figure 3

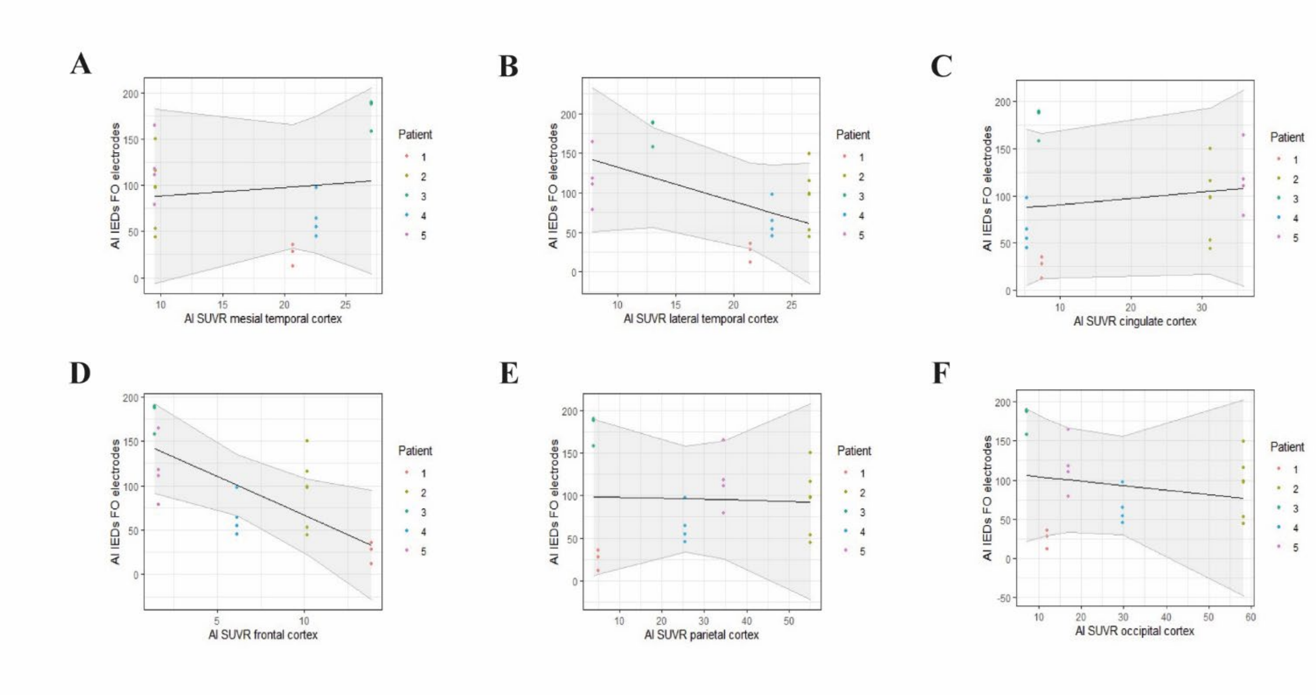

Supplementary Figure 3 Scatterplots of the AI of the IEDs detected on FO electrodes versus AI of the ^{18}F-MK6240 SUVR in different VOIs. Each point represents one overnight EEG recording. (**A)** Mesial temporal cortex; (**B)** Lateral temporal cortex; (**C)** Cingulate cortex; (**D)** Frontal cortex; (**E)** Parietal cortex; (**F)** Occipital cortex. Abbreviations: AI = Asymmetry index; FO = Foramen ovale; IEDs = Interictal epileptiform discharges; SUVR = Standardized uptake value ratio; VOI = Volume-of-interest.

3.4 Supplementary Figure 4

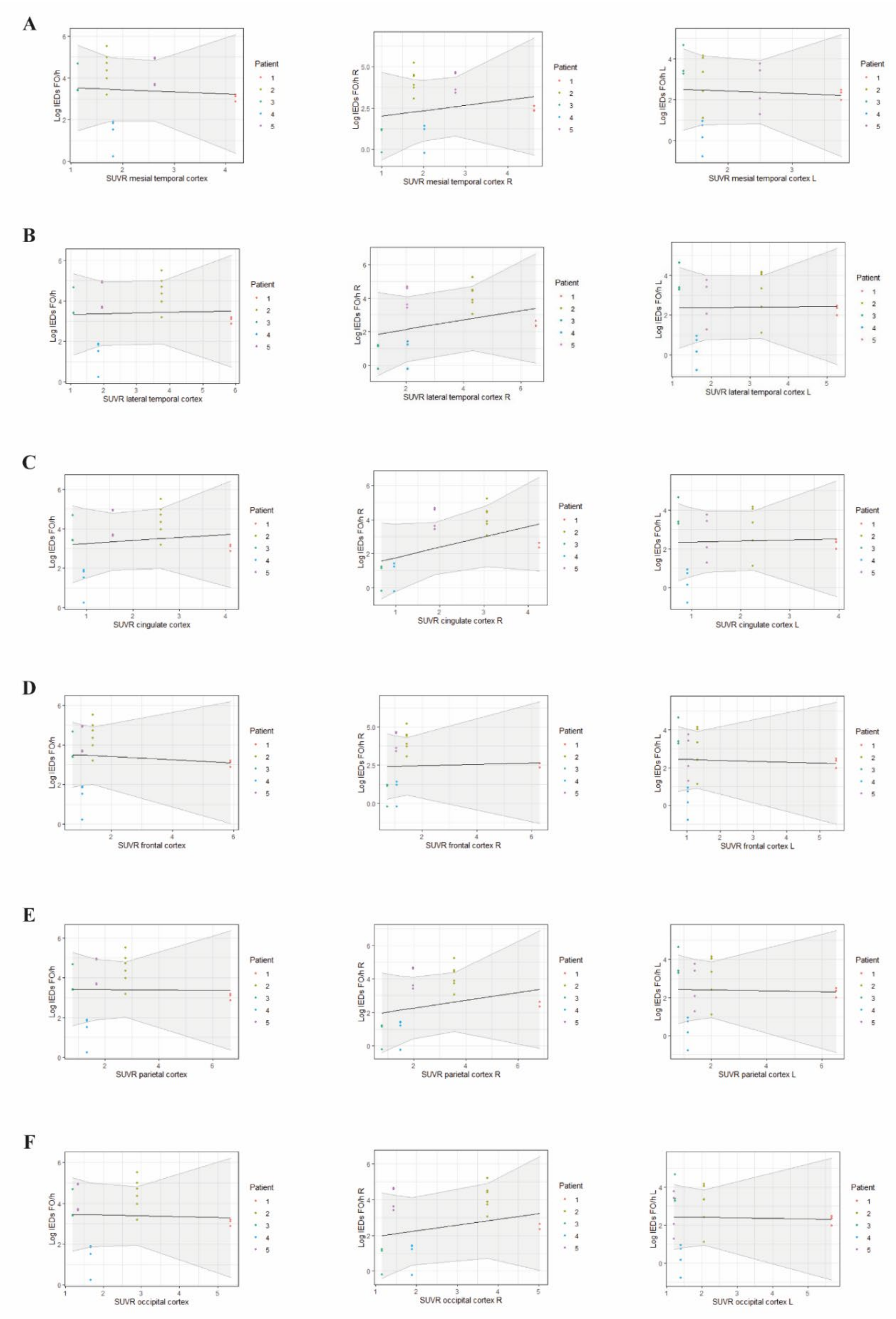

Supplementary Figure 4 Scatterplots of the IEDs/h detected on FO electrodes versus SUVR in different VOIs. In the left column the IEDs/h detected on bilateral FO electrodes versus SUVR of the bilateral VOIs; in the middle column the IEDs/h on the right FO electrode (FO R) versus SUVR of the VOIs in the right hemisphere; in the right column the IEDs/h on the left FO electrode (FO L) versus the SUVR of the VOIs in the left hemisphere. The plots show the natural logarithm of the IEDs/h detected on FO electrodes. Each point represents one overnight EEG recording. (**A**) Mesial temporal cortex; (**B**) Lateral temporal cortex; (**C**) Cingulate cortex; (**D**) Frontal cortex; (**E**) Parietal cortex; (**F**) Occipital cortex. Abbreviations: FO = Foramen ovale; IEDs/h = Interictal epileptiform discharges per hour; SUVR = Standardized uptake value ratio; VOI = Volume-of-interest.

4. References

1. Scheuer ML, Bagic A, Wilson SB. Spike detection: Inter-reader agreement and a statistical Turing test on a large data set. *Clin Neurophysiol.* 2017;128(1):243-250.

2. Reus EEM, Cox FME, Van Dijk JG, Visser GH. Automated spike detection: Which software package? *Seizure.* 2022;95:33-37.

3. Kural MA, Duez L, Sejer Hansen V*, et al*. Criteria for defining interictal epileptiform discharges in EEG: A clinical validation study. *Neurology.* 2020;94(20):e2139-e2147.

4. Hirsch LJ, Fong MWK, Leitinger M*, et al*. American Clinical Neurophysiology Society's Standardized Critical Care EEG Terminology: 2021 Version. *J Clin Neurophysiol.* 2021;38(1):1-29.
